# Human Bone‐Derived Endothelial Cells Mediate Bone Regeneration via Distinct Expression of KIT Ligand

**DOI:** 10.1002/advs.202414194

**Published:** 2025-06-23

**Authors:** Xiang Li, Hwan D. Kim, Allen C. Luo, Liyan Gong, Yonglin Zhu, Chin Nien Lee, Xuechong Hong, Christopher L. Sudduth, Michal Ad, Young‐Hyeon An, Mihn Jeong Park, Do‐Gyoon Kim, Arin K. Greene, Bonnie L. Padwa, Nathaniel S. Hwang, Ruei‐Zeng Lin, Juan M. Melero‐Martin

**Affiliations:** ^1^ Department of Cardiac Surgery Boston Children's Hospital Boston MA 02115 USA; ^2^ Department of Surgery Harvard Medical School Boston MA 02115 USA; ^3^ Department of Polymer Science and Engineering Korea National University of Transportation Chungju 27469 Republic of Korea; ^4^ Department of Pathology and Laboratory Medicine Epigenetics Institute Perelman School of Medicine University of Pennsylvania Philadelphia PA 19104 USA; ^5^ Department of Plastic and Oral Surgery Boston Children's Hospital Boston MA 02115 USA; ^6^ School of Chemical and Biological Engineering BioMAX Institute Institute of Chemical Processes Institute of Bioengineering Seoul National University Seoul 08826 Republic of Korea; ^7^ Present address: Division of Orthodontics College of Dentistry The Ohio State University Columbus OH 43210 USA; ^8^ Present address: Harvard Stem Cell Institute Cambridge MA 02138 USA

**Keywords:** angiocrine factors, bone regeneration, endothelial cells, osteogenic differentiation, vascular niche

## Abstract

Effective bone regeneration remains a significant challenge in surgical practice, particularly due to the limitations associated with autologous bone grafting, such as donor site morbidity and limited bone availability. This study investigated the potential of human bone‐derived endothelial cells (b‐ECs) in mediating bone regeneration, especially in conjunction with bone marrow‐derived mesenchymal stem cells (bm‐MSCs). It is demonstrated that b‐ECs retain unique osteoinductive properties post‐isolation, crucial for promoting bone formation in vivo. Utilizing ectopic and orthotopic xenograft models in immunodeficient mice, these findings revealed that the synergistic interaction of b‐ECs and bm‐MSCs induced rapid and substantial bone formation, highlighting the therapeutic potential of b‐ECs in bone repair strategies. The distinct expression of KIT ligand (KITLG) in b‐ECs emerged as a key factor in these processes. KITLG expression by b‐ECs facilitated the recruitment of c‐Kit+/CD34+ hematopoietic progenitor cells to the osteovascular niche, leading to robust osteogenic differentiation of bm‐MSCs, a process regulated by Notch signaling. Moreover, inducing KITLG expression in non‐bone‐derived endothelial cells conferred similar osteoinductive capabilities. These findings not only enhance the understanding of the intricate interplay between vascular and bone tissues but also open avenues for developing innovative cell‐based approaches for bone regeneration therapy.

## Graphical abstract

Bone‐specific endothelial cells (b‐ECs) uniquely express KITLG, which recruits c‐Kit+/CD34+ hematopoietic progenitors (HPCs) to the osteovascular niche, initiating a cascade that culminates in the osteogenic differentiation of bone marrow‐derived mesenchymal stem cells (bm‐MSCs) and subsequent ossification. This process underscores the potential of b‐ECs to enhance bone regeneration and repair by establishing an osteoinductive microenvironment.

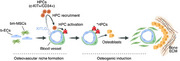

## 1 Introduction

Regeneration of bone defects remains a significant challenge in surgical practice. Annually, over three million musculoskeletal procedures are performed in the United States, with approximately half involving the repair of bone defects.^[^ ^1^ ^]^ Autologous bone grafting is the method of choice for bone repair due to its reduced risk of infection, enhanced osseointegration, and growth compatibility with the patient. However, the applicability of this method is constrained by the limited availability of donor bone and the morbidity associated with bone harvesting.^[^ ^2^, ^3^ ^]^ These limitations are particularly acute in the pediatric population, where the scarcity of transplantable bone further complicates the situation.^[^ ^4^, ^5^ ^]^ Consequently, there is a pressing clinical need for novel approaches to bone repair.^[^ ^6^ ^]^

In recent years, the quest for effective bone regeneration strategies has intensified, focusing on various modalities such as bone grafts, biocompatible scaffolds, growth factors, and, notably, stem cells. Among these, exploiting the osteogenic potential of bone marrow‐derived mesenchymal stem cells (bm‐MSCs) has been a long‐standing focus in regenerative medicine for bone repair.^[^ ^7^, ^8^ ^]^ The clinical adoption of MSC‐based therapies holds substantial promise due to their potential to circumvent donor‐site morbidity associated with autologous bone grafting, mitigate the risk of graft resorption, and provide a renewable source of bone graft substitutes. However, translating these MSC‐based approaches into clinical practice faces significant challenges. These include the efficient engraftment of human bm‐MSCs and the realization of their osteogenic potential in a physiological in vivo environment, which remains complex and unresolved.

Establishing stable engraftment of bm‐MSCs in vivo has been a persistent challenge in regenerative medicine.^[^ ^7^, ^8^ ^]^ Accumulating research reveals that MSCs are typically located within vascular niches in postnatal tissues, suggesting an intrinsic relationship with vascular structures.^[^ ^9^ ^]^ Indeed, the interplay of paracrine signals between endothelial cells (ECs) and MSCs plays a central role in the functionality of these niches.^[^ ^10^, ^11^, ^12^ ^]^ However, when isolated for therapeutic purposes, MSCs lose their proximal interaction with ECs, thereby disrupting this critical crosstalk. Our previous studies showed the dependence of human bm‐MSC osteogenic potential on maintaining their proximity to ECs in vivo.^[^ ^13^ ^]^ We demonstrated that co‐transplantation with ECs could provide essential paracrine trophic factors like PDGF‐BB, crucial for the robust engraftment of bm‐MSCs as perivascular cells. Furthermore, we showed that the disruption of these EC‐derived factors significantly impairs the osteogenic differentiation capacity of bm‐MSCs.^[^ ^13^ ^]^ These insights contributed to a better understanding of the engraftment process, marking a step forward in enhancing the efficacy of MSC‐based regenerative strategies.

An additional challenge in regenerative bone therapy is regulating the osteogenic differentiation of the engrafted bm‐MSCs in vivo. Conventionally, this process has relied on the application of exogenous factors, such as recombinant human bone morphogenetic protein‐2 (BMP‐2) or parathyroid hormones.^[^ ^14^, ^15^ ^]^ However, these approaches often result in a mineralized matrix that lacks vascularization and fails to integrate effectively with the existing bone tissue. Moreover, the use of high doses of BMP‐2 has been associated with undesirable effects, including heterotopic bone formation, raising concerns about its clinical applicability.^[^ ^16^, ^17^, ^18^ ^]^ In vivo studies in mice have established the crucial role of the bone endothelium in driving bm‐MSC osteogenesis during development.^[^ ^19^, ^20^ ^]^ However, the translation of these findings to human physiology remains underexplored. Moreover, it is unknown whether postnatal bone ECs (b‐ECs) preserve their osteoinductive capabilities ex vivo, a question vital to their potential therapeutic value in driving osteogenic differentiation in a clinical setting.

In this study, we demonstrate that b‐ECs preserve their osteoinductive capabilities post‐isolation. We show that when b‐ECs and bm‐MSCs are utilized together in xenograft models, they synergistically induce bone formation, underscoring the potential of b‐ECs in bone repair strategies. Furthermore, we elucidate that this osteoinductive property is intrinsic to b‐ECs and is primarily attributed to their specific expression of KITLG, a pivotal mediator in this process. This KITLG expression in b‐ECs facilitates the recruitment of c‐Kit+/CD34+ hematopoietic progenitor cells (HPCs) into the implanted grafts, which in turn mediates the osteogenic differentiation of bm‐MSCs via the Notch signaling pathway. Notably, our findings also reveal that by inducing KITLG expression in non‐bone‐derived ECs, these cells can acquire similar osteoinductive abilities. Collectively, our findings not only reveal the critical role of b‐ECs in regulating osteogenic differentiation but also suggest that these tissue‐specific endothelial characteristics could be leveraged for future therapeutic strategies in bone regeneration.

## 2 Results

### 2.1 Human Bone‐Derived ECs Exhibit Osteoinductive Properties In Vivo

In an effort to elucidate the osteogenic potential of b‐ECs, we obtained de‐identified human iliac crest trabecular bone samples. The isolation of b‐ECs entailed an enzymatic digestion regimen utilizing collagenase/dispase, followed by the enrichment of CD31+ cells (Figure S1A, Supporting Information). Flow cytometric analysis of the freshly isolated b‐ECs revealed a composition of ≈40% CXCR4+/CD31+ arteriole ECs and 60% CXCR4‐/CD31+ sinusoid ECs (Figure S1B, Supporting Information), consistent with previous findings.^[^ ^11^ ^]^ Upon expansion in culture, the prevalence of these arteriole b‐ECs increased to nearly 95% (Figure S1B,Supporting Information), indicating their preferential propagation in vitro over their sinusoidal counterparts.

**Figure 1:**
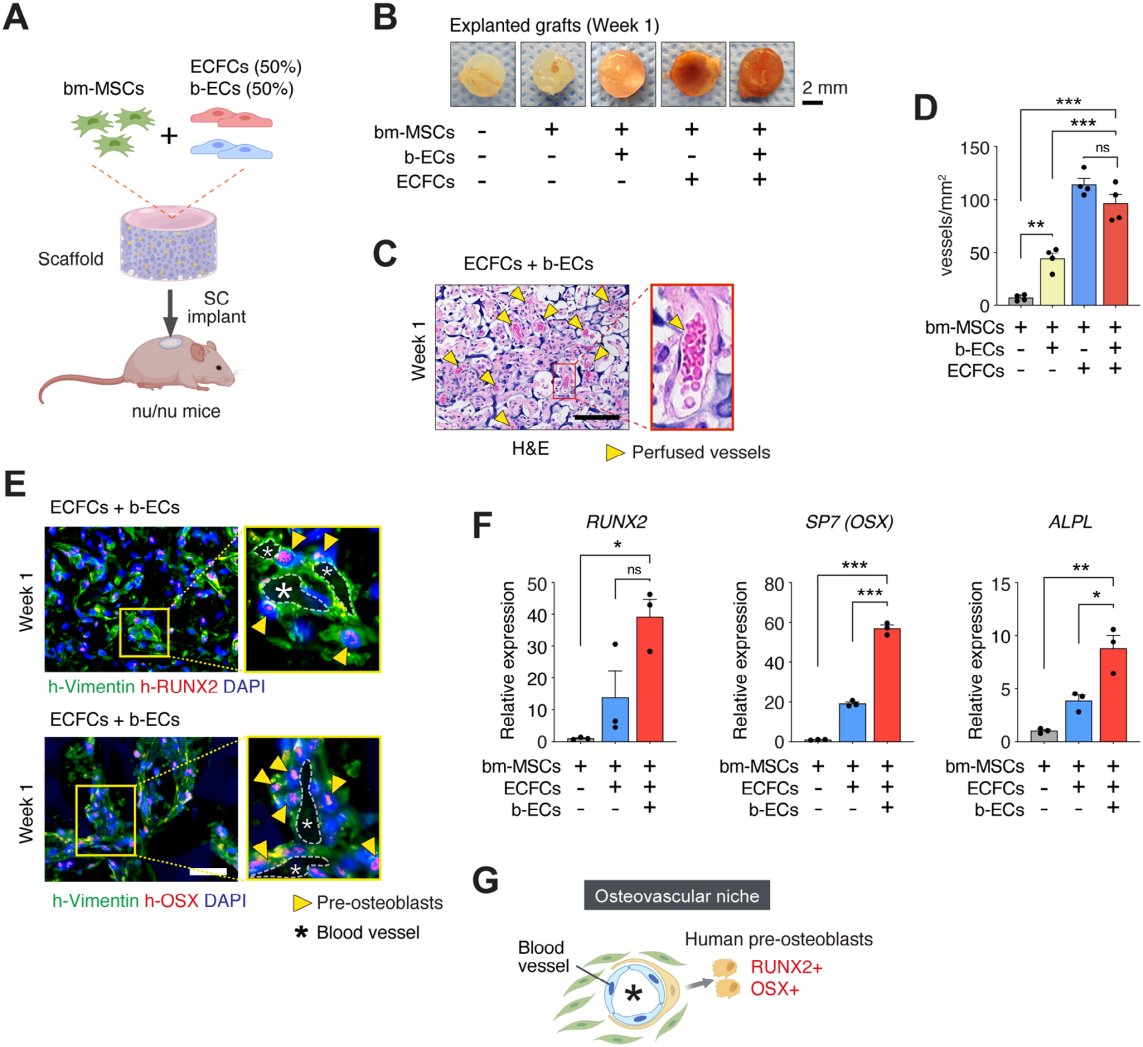
Osteovascular niche formation in biomimetic scaffold implants with b‐ECs. A) Diagram illustrating the subcutaneous (SC) implantation of a scaffold with bm‐MSCs and ECFCs alone or in combination with b‐ECs into nu/nu mice. B) Macroscopic images of grafts containing bm‐MSCs and different combinations of ECs explanted at Week 1. Scale bar: 2 mm. C) H&E staining of grafts with b‐ECs at Week 1, showing perfused blood vessels (yellow arrowheads). Scale bar: 100 µm. D) Quantification of perfused microvessel density in explanted grafts (*n* = 4; ***p < 0.001). E) Immunofluorescence staining for human‐specific h‐Vimentin (green) and RUNX2 (red, upper panel) or OSX (red, lower panel) in grafts with b‐ECs at Week 1, indicating the presence of pre‐osteoblasts (yellow arrowheads) and blood vessels (white asterisks). Scale bar: 100 µm. F) qPCR analysis of osteogenic markers *RUNX2*, *SP7* (OSX), and *ALPL* in grafts at Week 1 (*n* = 3; *p < 0.05, **p < 0.01, ***p < 0.001). G) Schematic representation of an osteovascular niche within a graft containing b‐ECs. All data are mean ± s.e.m. *n* are biological replicates (D,F). Statistics are one‐way ANOVA with Bonferroni's post‐test analysis (D, F). Panels A and G were partially created with BioRender.com.

Further characterization confirmed that our cultured b‐ECs retained endothelial characteristics, as evidenced by their morphological attributes and the expression of endothelial markers such as CD31/*PECAM1*, VE‐Cadherin/*CDH5*, *VEGFR‐2*, and vWF, while not expressing mesenchymal (CD90/*THY1*, CD140b/*PDGFRB*) or hematopoietic (CD45) cell markers (Figure S1C–E, Supporting Information), with an average purity exceeding 95%.

Next, we examined the capacity of b‐ECs to form vascular networks in vivo and their role in the osteogenic differentiation of bone marrow‐derived MSCs (bm‐MSCs). We utilized a biomimetic scaffold we previously developed for rapid and sustained ossification. This scaffold, composed of chondroitin sulfate cryogel with whitlockite calcium phosphate nanoparticles, is designed to facilitate the ossification process following the osteogenic differentiation of bm‐MSCs.^[^ ^21^, ^22^, ^23^ ^]^ We seeded these scaffolds with cells resuspended in Matrigel and implanted them subcutaneously into nude mice (**Figure** 1A). We initially compared grafts containing bm‐MSCs alone, bm‐MSCs with b‐ECs, and bm‐MSCs with ECFCs. However, we observed a significant disparity in vascularization, with grafts containing bm‐MSCs and ECFCs showing much higher vascular density (≈100 vessels mm^−^^2^) compared to those with bm‐MSCs and b‐ECs (<50 vessels mm^−^^2^) (Figure 1B–D). To address this disparity and maintain the high level of vascularization necessary for successful graft integration, we introduced a new group: bm‐MSCs with a 1:1 mixture of b‐ECs and ECFCs. This combination allowed us to study the effect of b‐ECs without compromising the vascularization of the graft and its engraftment. Indeed, grafts with the mixed EC population demonstrated vascularization levels comparable to those with ECFCs alone (Figure 1D), thereby optimizing the osteovascular outcomes in our biomimetic scaffold implants.

Furthermore, grafts with b‐ECs uniquely induced the expression of osteogenic differentiation markers compared to those with only ECFCs or no ECs. After 1 week, only the b‐EC grafts showed a significant increase in cells expressing *RUNX2*, osterix (*OSX/SP7*), and *ALPL*, indicating the onset of an osteovascular niche and the initiation of bm‐MSC differentiation into osteoblasts (Figure 1E–G). This finding highlights the distinct ability of b‐ECs to establish an osteovascular niche and promote early‐stage osteogenic differentiation of bm‐MSCs.

### 2.2 Enhanced Ossification in Ectopic Grafts Vascularized by b‐ECs

**Figure 2:**
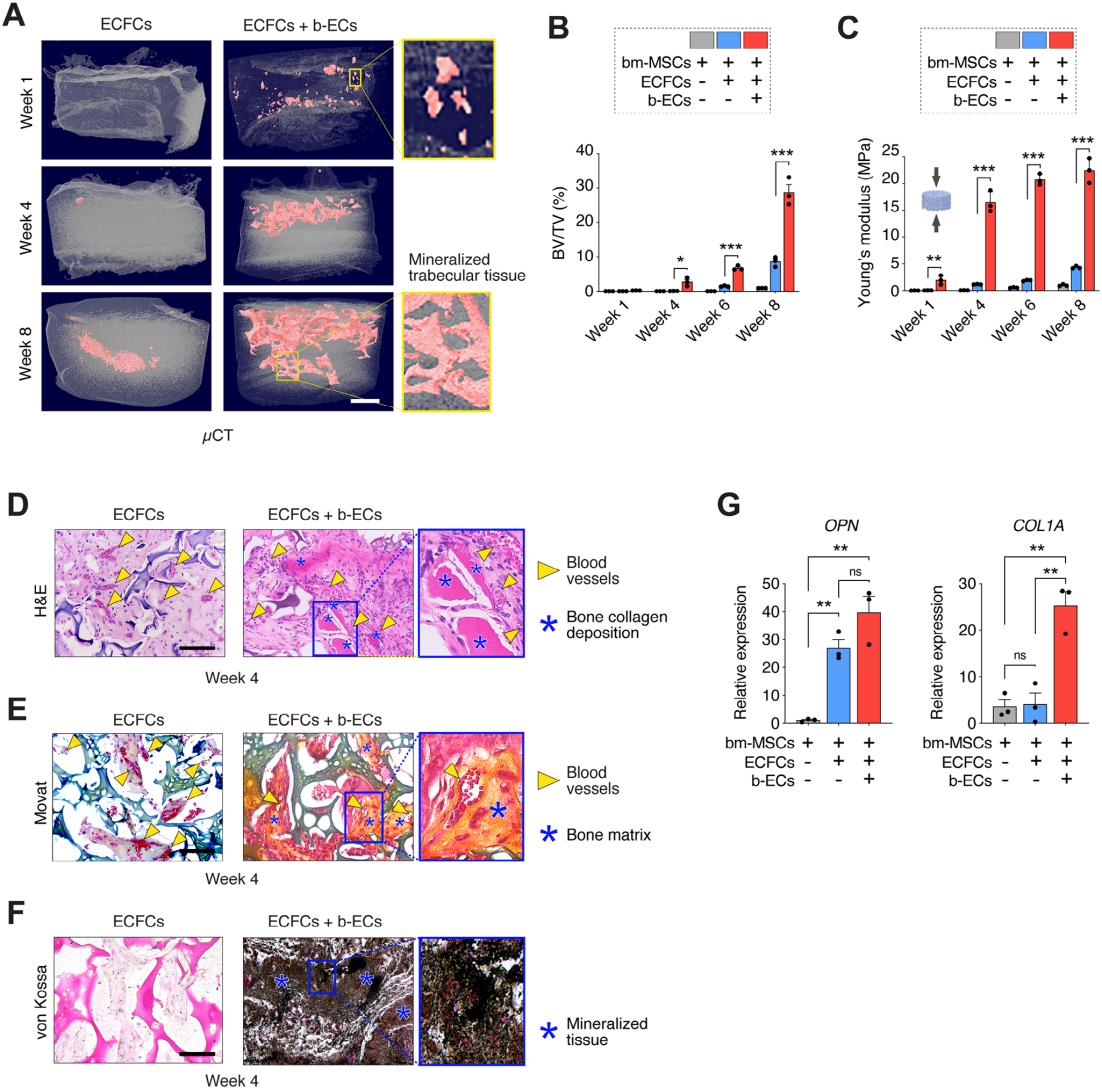
Bone‐derived ECs induce robust ossification of subcutaneous ectopic grafts. A) µCT images showing mineralized tissue in red at Weeks 1, 4, and 8 post‐implantation, with insets detailing the mineralized regions. Scale bar: 1 mm. B) Quantification of bone volume fraction (BV/TV) over the implantation period (*n* = 3; *p < 0.05, ***p < 0.001). C) Compressive Young's modulus of scaffolds over time indicating increased mechanical strength in b‐EC‐containing grafts (*n* = 3; **p < 0.01, ***p < 0.001). D) H&E staining illustrates the presence of blood vessels (yellow arrowheads) and bone collagen deposition (blue asterisks) at Week 4. Scale bar: 100 µm. E) Movat Pentachrome staining at Week 4 shows blood vessels (yellow arrowheads) and mineralized bone matrix (blue asterisks). Scale bar: 100 µm. F) Von Kossa staining at Week 4 reveals mineralized bone tissue (black areas). Scale bar: 100 µm. G) qPCR results for bone regeneration markers *OPN* and *COL1A* at Week 6, indicating enhanced expression in b‐EC‐containing grafts (n = 3; *p < 0.05, **p < 0.01). All data are mean ± s.e.m. *n* are biological replicates (B, C, G). Statistics are one‐way ANOVA with Bonferroni's post‐test analysis (B, C, G).

To further elucidate the ability of b‐ECs to induce ossification at an ectopic site, we performed micro‐computed tomography (µCT) analysis on grafts containing b‐ECs. As early as 1 week, these grafts demonstrated substantial mineralization, with an interconnected trabecular network becoming evident by Week 8 (**Figure** **2**A,B; Figure S2A,B, and Video S1, Supporting Information). In contrast, grafts without b‐ECs exhibited minimal mineralization (Figure 2B). The enhanced mineralization in b‐EC‐containing grafts also translated into superior mechanical properties, as evidenced by significantly higher compressive strength (Young's modulus) over time (Figure 2C; Figure S2C,D, Supporting Information).

Histochemical analyses at 4 weeks further confirmed the superior bone collagen/matrix deposition in grafts with b‐ECs, as shown by H&E and Movat staining (Figure 2D,E; Figure S3, Supporting Information). Von Kossa staining corroborated the extensive calcification within these grafts (Figure 2F). Additionally, gene expression analyses at 6 weeks revealed elevated levels of osteogenic and ossification markers, such as *OPN* and *COL1A*, in grafts seeded with b‐ECs (Figure 2G).

Notably, the emergence of pre‐osteoblasts and subsequent ossification in these ectopic grafts occurred without the addition of exogenous growth factors like BMP‐2, which are commonly used in bone tissue engineering. Importantly, our previous findings demonstrated that the scaffold alone does not induce osteogenic differentiation of bm‐MSCs,^[^ ^21^ ^]^ further underscoring the inherent osteoinductive property of b‐ECs in directing bm‐MSC differentiation within the biomimetic scaffolds. These results demonstrate that the presence of b‐ECs significantly enhances ossification and creates an environment conducive to bm‐MSC osteogenic differentiation, independent of exogenous osteoinductive factors.

### 2.3 Robust Orthotopic Ossification of Critical‐Size Calvarial Defects

Our ectopic transplantation experiments demonstrated that b‐ECs can establish osteovascular niches at non‐bone sites, independent of endogenous bone regenerative signals. To further validate the ossification capability of b‐ECs, we transitioned to an orthotopic model of nonhealing critical‐sized calvarial defects,^[^ ^24^ ^]^ which is widely recognized for evaluating the inherent regenerative potential of cells in non‐load‐bearing bones, such as the calvarium. This provides a platform to test the unique advantages of b‐ECs in bone regeneration.

**Figure 3:**
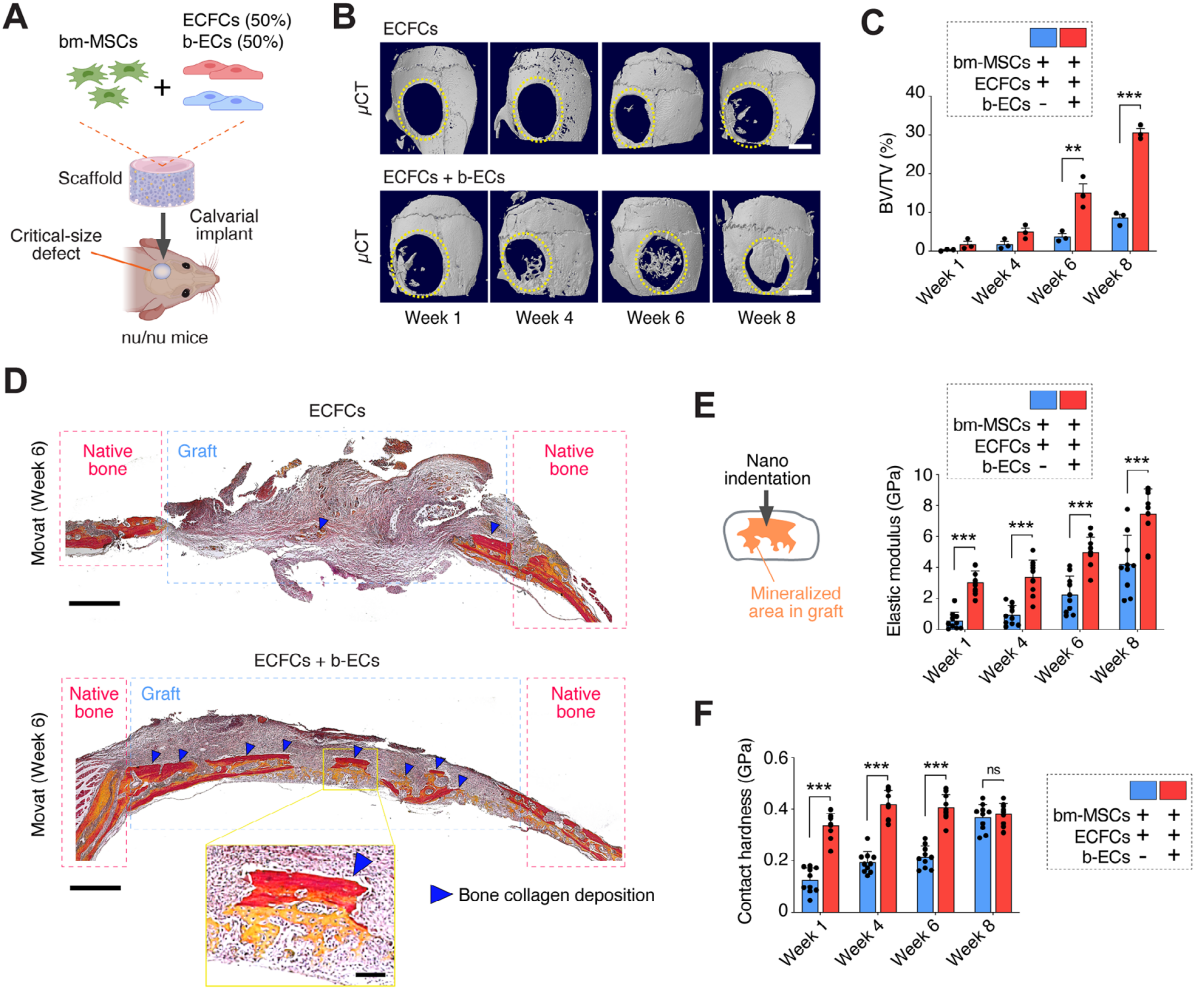
Bone‐derived ECs induce robust ossification of calvarial bone defects. A) Illustration showing the implantation of a scaffold with bm‐MSCs and ECFCs, with or without b‐ECs, into calvarial defects of nu/nu mice. B) µCT images at 1, 4, 6, and 8 weeks post‐implantation, with the original defect outlined by yellow dotted lines. Scale bar: 2 mm. C) Bone volume fraction (BV/TV) quantification across the implantation period (*n* = 3; **p < 0.01, ***p < 0.001). D) Movat Pentachrome‐stained cross sections of grafts at Week 6; blue dotted lines demarcate graft regions, and blue arrowheads indicate bone collagen deposition. Scale bar: 600 µm; inset scale bar: 100 µm. E) Elastic modulus and F) contact hardness of mineralized areas within grafts measured at 1, 4, 6, and 8 weeks post‐implantation (*n* = 7‐10; ***p < 0.001; n.s., not significant). All data are mean ± s.e.m. *n* are biological replicates (C, E, F). Statistics are one‐way ANOVA with Bonferroni's post‐test analysis (C, E, F). Panels A was partially created with BioRender.com.

We surgically generated critical‐sized (4‐mm) full‐thickness calvarial bone defects in adult nude mice and implanted scaffolds laden with bm‐MSCs, alongside either ECFCs alone or a 1:1 combination of ECFCs and b‐ECs (**Figure** **3**A). Quantitative µCT scans revealed substantial mineralized tissue formation exclusively in grafts containing b‐ECs over an eight‐week period (Figure 3B,C; Figure S4C, Supporting Information). By week eight, nearly complete mineralization of the defects was observed in the b‐EC grafts (Figure 3B). Histological evaluations further confirmed the presence of an organized mineralized bone matrix (Figure 3D) and a significant enrichment of human‐specific osteocalcin (OCN)+ cells in grafts seeded with b‐ECs (Figure S4A,B, Supporting Information). Mechanical analysis using nanoindentation revealed a progressive improvement in the material properties of these mineralized zones, significantly surpassing those of grafts containing only ECFCs (Figure 3E,F).

Together, these findings emphasize the ability of b‐ECs to promote orthotopic bone regeneration, outperforming generic ECFCs. Although this model does not assess weight‐bearing bone, it demonstrates the distinct advantage of bone‐specific ECs in enhancing bone regeneration.

### 2.4 Differentially Expressed KITLG in Human b‐ECs Mediates Osteogenesis In Vivo

**Figure 4:**
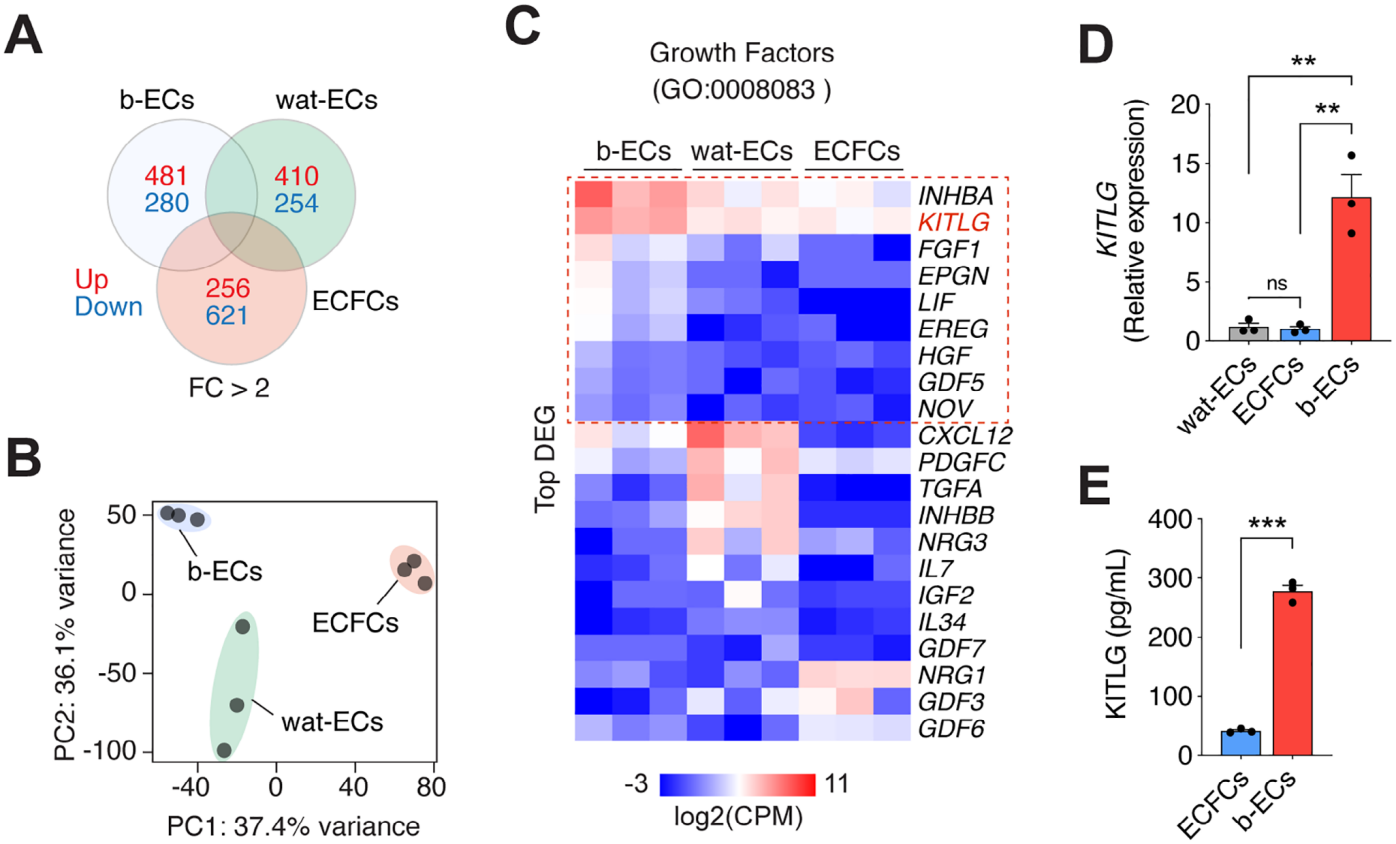
Differential expression of KITLG in human b‐ECs. A) Venn diagram showing the number of up‐ and down‐regulated genes (FC > 2; adjusted *p* < 0.05) in b‐ECs, wat‐ECs, and ECFCs. Samples for each group are from 3 individual subjects. B) PCA of global gene expression profiles distinguishing b‐ECs, wat‐ECs, and ECFCs. C) Heatmap of differentially expressed growth factor genes (GO:0 0 08083) with clusters enriched in b‐ECs outlined by the red dotted box. D) Relative *KITLG* mRNA expression in b‐ECs, wat‐ECs, and ECFCs measured by qPCR (*n* = 3; **P < 0.01). E) ELISA quantification of KITLG protein secreted by b‐ECs and ECFCs in 72‐h conditioned media (*n* = 3; ***p < 0.001). All data are mean ± s.e.m. *n* are biological replicates (D, E). Statistics are one‐way ANOVA with Bonferroni's post‐test analysis (D) and unpaired two‐tailed Student's t‐tests (E).

To elucidate the role of b‐ECs in osteogenesis, we conducted bulk RNA‐seq analysis comparing the gene expression profiles of ECs derived from human bone tissue (b‐ECs), white adipose tissue (wat‐ECs), and umbilical cord blood (ECFCs). While the transcriptional analysis revealed overall similarities among these EC types, reflecting their common endothelial lineage (**Figure** **4**A), principal component analysis highlighted distinct clusters of differentially expressed genes in each group, suggesting unique functional roles (Figure 4B).

We specifically examined genes involved in growth factor activity (gene ontology term GO:0 0 08083; 162 genes). Among the top genes identified in b‐ECs were *KITLG* and *INHBA*, both upregulated compared to ECFCs and wat‐ECs (Figure 4C). While INHBA is implicated in various cellular processes, including tissue remodeling, inflammation, and fibrosis,^[^ ^25^, ^26^ ^]^ our focus on KITLG was driven by its well‐documented role in maintaining hematopoietic niches,^[^ ^27^, ^28^, ^29^, ^30^ ^]^ particularly in bone tissue. Also, the upregulation of KITLG in b‐ECs was confirmed at both the mRNA and protein levels (Figure 4D,E). This finding aligns with previous studies in murine models, which have also demonstrated selective *KITLG* expression in bone arteriole ECs.^[^ ^28^ ^]^ Nevertheless, although KITLG is well‐known for its role in maintaining hematopoietic niches, its direct involvement in osteogenesis has not been previously established.

**Figure 5:**
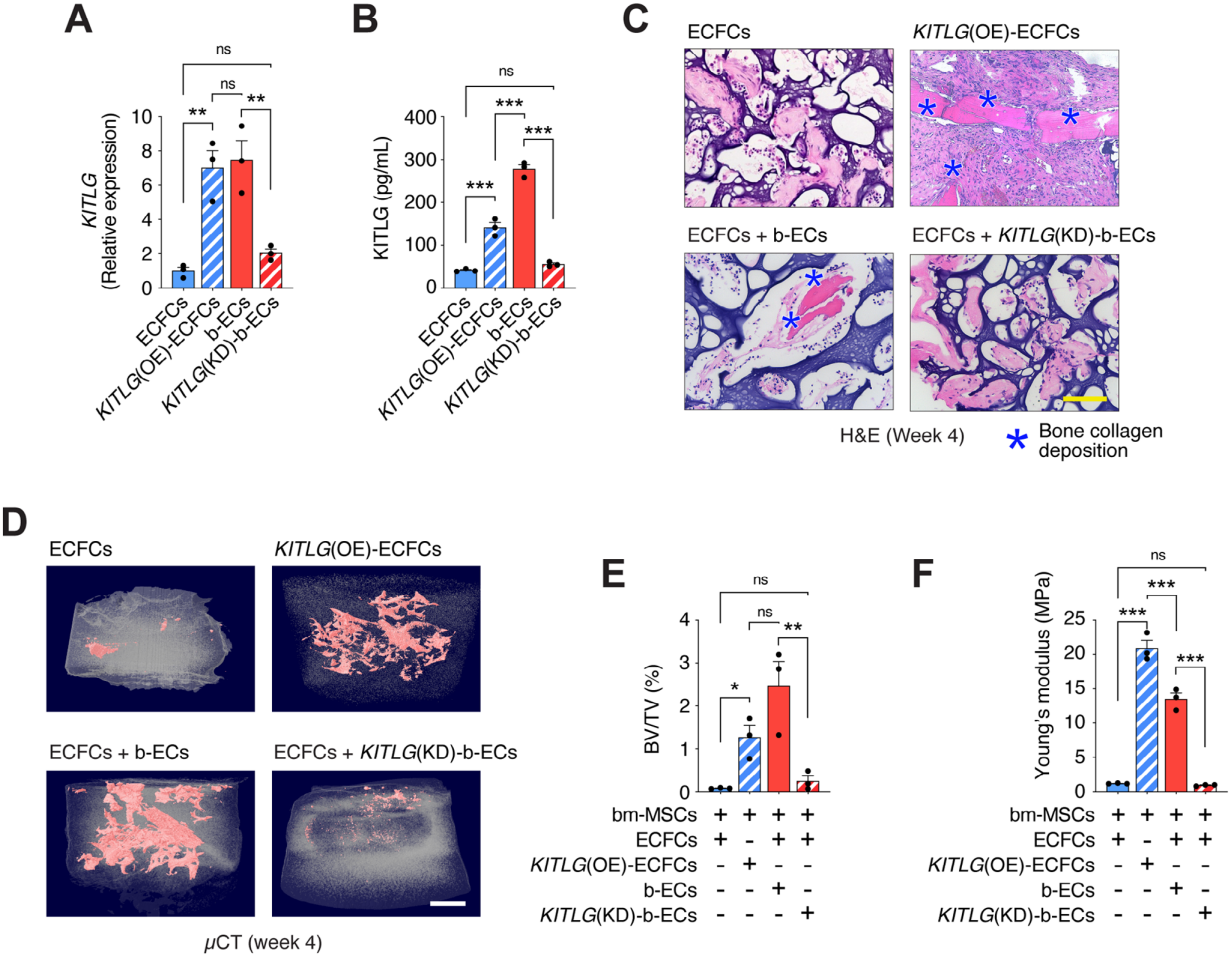
KITLG expression in ECs mediates ossification. A) qRT‐PCR validation showing effective knockdown of *KITLG* in b‐ECs (KITLG(KD)) and overexpression in ECFCs (KITLG(OE)) compared to unmodified controls (*n* = 3; **p < 0.01). B) ELISA quantification of secreted KITLG protein in conditioned media, confirming that protein‐level modulation mirrors the transcriptional changes observed in (A) (*n* = 3; ***p < 0.001). C–F) Subcutaneous implantation of scaffolds with bm‐MSCs and the indicated EC group into nu/nu mice. C) H&E staining of grafts at 4 weeks post‐implantation. Blue asterisks indicate areas of bone collagen deposition. Scale bar: 100 µm. D) µCT images of grafts at 4 weeks showing the extent of mineralized tissue. Scale bar: 1 mm. E) Quantification of bone volume fraction (BV/TV) at 4 weeks (*n* = 3; *p < 0.05, **P < 0.01). F) Compressive Young's modulus of grafts at 4 weeks, reflecting the mechanical properties of the grafts in response to KITLG expression changes (*n* = 3; ***p < 0.001). All data are mean ± s.e.m. *n* are biological replicates (A, B, E, F). Statistics are one‐way ANOVA with Bonferroni's post‐test analysis (A, B, E, F).

To investigate KITLG's specific effect on osteogenesis, we conducted loss‐of‐function and gain‐of‐function studies by silencing *KITLG* in b‐ECs (KITLG(KD)‐b‐ECs) and overexpressing it in ECFCs (KITLG(OE)‐ECFCs). Successful knockdown and overexpression of KITLG were confirmed at both the mRNA (qRT‐PCR) and protein (ELISA) levels (**Figure** **5**A,B). Scaffolds containing bm‐MSCs and these modified ECs were implanted as ectopic grafts, and ossification was evaluated after four weeks. Histological and µCT analyses showed that silencing *KITLG* in b‐ECs completely inhibited mineralization and bone matrix formation (Figure 5C,D), whereas overexpressing *KITLG* in ECFCs resulted in significant mineralization and increased bone collagen deposition, similar to grafts with unmodified b‐ECs (Figure 5C–E). Full‐graft low‐magnification images with randomized high‐magnification selections are provided for each group (Figure S5, Supporting Information). Furthermore, the mechanical properties, particularly compressive Young's modulus, correlated with the observed mineralization patterns (Figure 5F).

To further clarify these findings, we conducted additional control experiments modulating KITLG expression in b‐ECs and ECFCs. Overexpression of KITLG in b‐ECs [KITLG(OE)‐b‐ECs] did not further enhance osteogenic gene expression or affect vascular network formation, suggesting that b‐ECs already express KITLG at or near a functional threshold (Figure S6, Supporting Information). Similarly, knockdown of KITLG in ECFCs [KITLG(KD)‐ECFCs], which endogenously express low KITLG levels, did not impair osteogenesis or angiogenesis (Figure S6, Supporting Information). These control experiments further validate the central role of KITLG in mediating the osteoinductive capacity of b‐ECs.

Taken together, these results suggest that KITLG expression is both necessary and sufficient for initiating osteogenic differentiation, irrespective of the presence of bone‐specific b‐ECs. Collectively, these findings indicate that KITLG is essential for b‐EC‐driven osteogenesis and can confer osteogenic potential to other EC types.

### 2.5 KITLG Modulates the Recruitment of Hematopoietic Progenitor Cells

**Figure 6:**
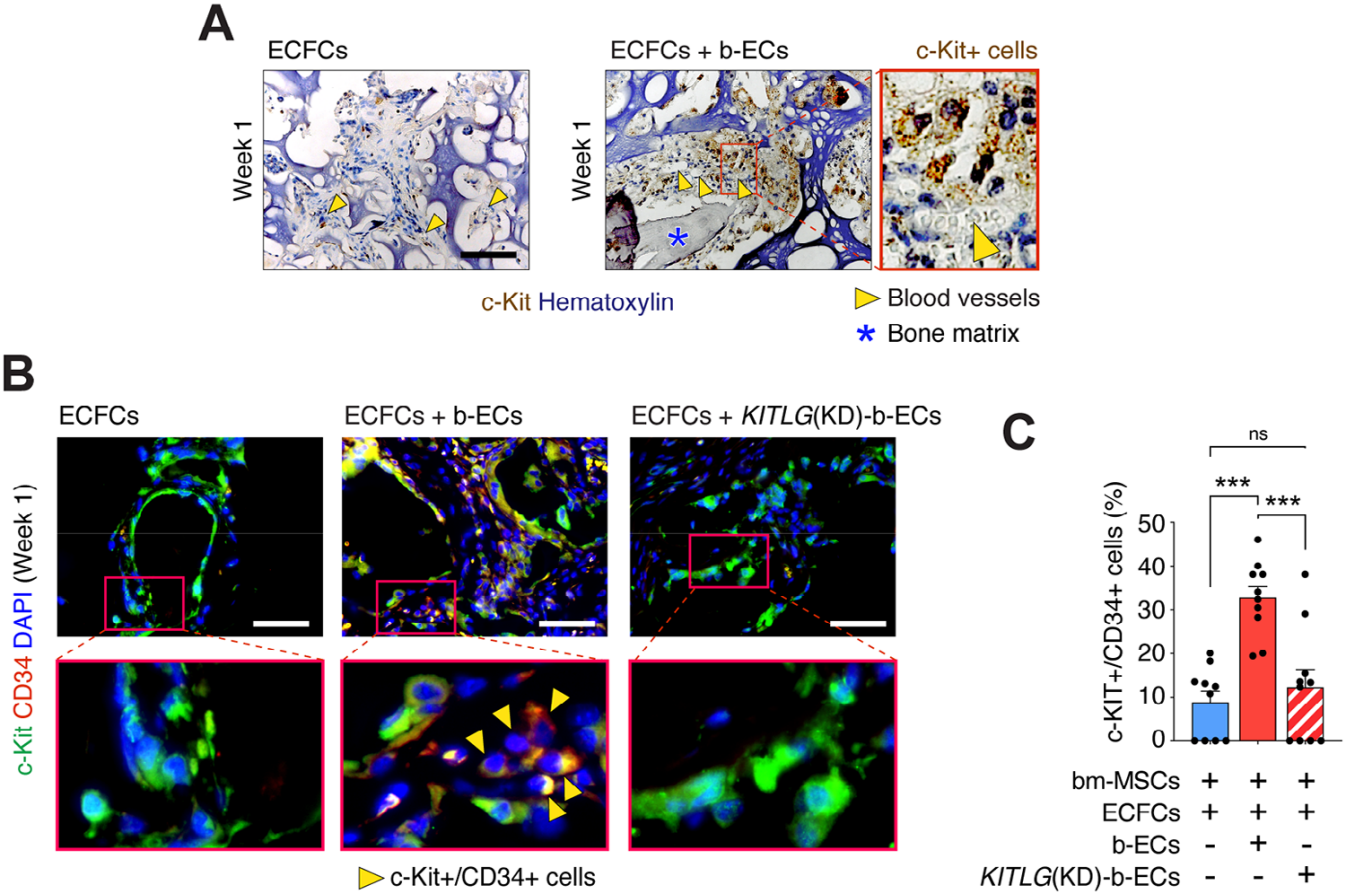
Enriched recruitment of c‐Kit+/CD34+ cells to grafts containing b‐ECs. Subcutaneous grafts containing bm‐MSCs and ECFCs alone or in combination with b‐ECs, implanted into nu/nu mice. A) Immunohistochemical staining depicting c‐Kit+ cell distribution at Week 1, with yellow arrowheads indicating blood vessels and blue asterisks denoting bone matrix. Scale bar: 100 µm. Inset shows a robust presence of c‐Kit+ cells recruited around blood vessels in a representative graft containing b‐ECs. B) Representative immunofluorescence images showing c‐Kit⁺ (green) and CD34⁺ (red) cell recruitment at Week 1 across all experimental groups: ECFCs alone, ECFCs + b‐ECs, and ECFCs + KITLG(KD)‐b‐ECs. Nuclei were counterstained with DAPI (blue). Yellow arrowheads indicate c‐Kit⁺/CD34⁺ double‐positive cells. Scale bar: 100 µm. C) Quantification of c‐Kit⁺ and CD34⁺ cells at 1 week (*n* = 10; ***P < 0.001). Data are mean ± s.e.m. *n* are histological fields from independent biological replicates. Statistics are one‐way ANOVA with Bonferroni's post‐test analysis.

Given that c‐Kit serves as the receptor for KITLG,^[^ ^31^ ^]^ we investigated the recruitment of c‐Kit+ cells in grafts containing b‐ECs. During the initial post‐implantation phase at Week 1, grafts containing b‐ECs exhibited a pronounced increase in the c‐Kit+ cell population near blood vessels compared to those seeded with ECFCs alone (**Figure** **6**A). Immunofluorescence staining further revealed that the majority of c‐Kit+ cells recruited to these grafts co‐expressed CD34, consistent with a hematopoietic progenitor cell (HPC) phenotype.^[^ ^32^, ^33^ ^]^ This recruitment was significantly higher in grafts with b‐ECs than in those containing either ECFCs or KITLG(KD)‐b‐ECs (Figure 6B,C). Inhibition of *KITLG* expression in b‐ECs led to a significant reduction in the recruitment of c‐Kit+ HPCs, underscoring the critical role of KITLG in mediating this process (Figure 6C).

These results confirm a direct correlation between KITLG expression in b‐ECs and the selective recruitment of c‐Kit+/CD34+ HPCs, highlighting the importance of KITLG in mobilizing progenitor cells to bone regenerative sites.

### 2.6 HPCs Mediate the Osteogenic Differentiation of bm‐MSCs

**Figure 7:**
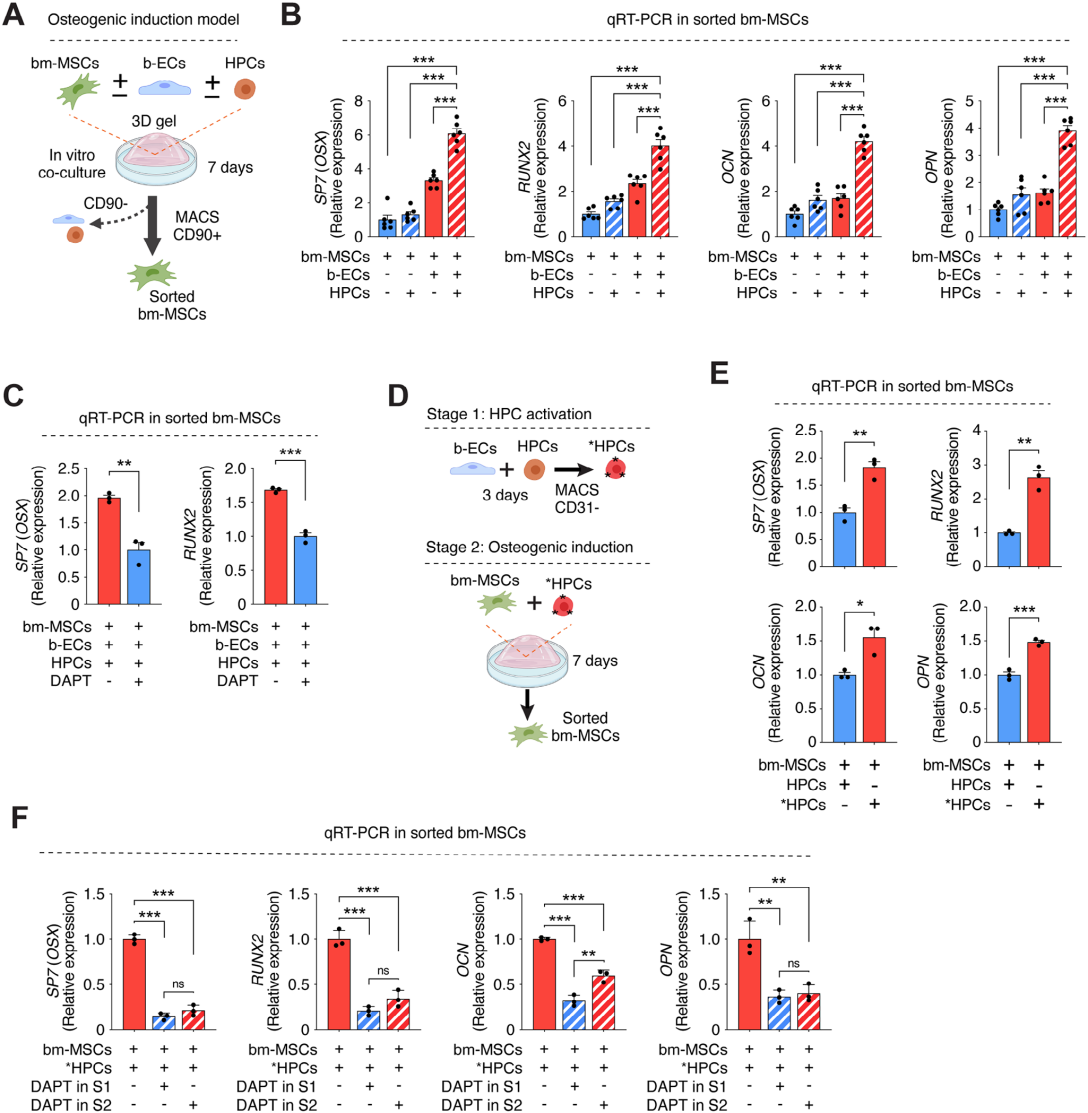
HPCs mediate osteogenic differentiation of bm‐MSCs through b‐EC interactions. A) Schematic of an in vitro model with bm‐MSCs co‐cultured in a 3D hydrogel with b‐ECs and HPCs for 7 days, followed by sorting for qPCR analysis. B) Osteogenic marker expression by qPCR in sorted bm‐MSCs under various culture conditions (*n* = 6; ***p < 0.001). C) Early osteogenic marker expression by qPCR in bm‐MSCs co‐cultured with b‐ECs and HPCs for 3 days, with and without Notch pathway inhibition by DAPT (*n* = 3; **p < 0.01, ***p < 0.001). D) Two‐stage in vitro model schematic: Stage 1 – HPC activation with b‐ECs for 3 days; Stage 2 – co‐culture of activated HPCs (termed *HPCs) with bm‐MSCs in a 3D hydrogel for 7 days before qPCR analysis. E) Osteogenic marker expression by qPCR in bm‐MSCs after co‐culture with either naïve or activated *HPCs (n = 3; *p < 0.05, **P < 0.01, ***p < 0.001). F) Osteogenic marker expression by qPCR in bm‐MSCs following co‐culture with activated *HPCs, with or without DAPT treatment during Stages 1 (S1) and 2 (S2) (*n* = 3; **p < 0.01, ***p < 0.001). All data are mean ± s.e.m. *n* are biological replicates (B, C, E, F). Statistics are one‐way ANOVA with Bonferroni's post‐test analysis (B, C, E, F). Panels A and D were partially created with BioRender.com.

To dissect the specific role of c‐Kit+ HPCs in the osteogenic differentiation of bm‐MSCs, we developed an in vitro model using Lin‐/c‐Kit+ HPCs isolated from human trabecular bone, alongside b‐ECs and bm‐MSCs (**Figure** **7**A). The colony‐forming capacity of the isolated c‐Kit+ HPCs, a hallmark of hematopoietic progenitor potential, was validated through standard methylcellulose assays (Figure S7, Supporting Information). Specifically, we co‐cultured bm‐MSCs with or without b‐ECs and c‐Kit+ HPCs in a 3D collagen/fibrin hydrogel scaffold and then examined the expression of osteogenic markers by qPCR in bm‐MSCs sorted from the co‐culture system. After seven days, we observed a significant upregulation of osteogenic markers—*SP7* (*OSX*), *RUNX2*, *OCN*, and *OPN*—in bm‐MSCs co‐cultured with both b‐ECs and c‐Kit+ HPCs (Figure 7B). In contrast, co‐cultures lacking either b‐ECs or c‐Kit+ HPCs did not induce the same level of osteogenic differentiation in bm‐MSCs, underscoring the necessity of both cell types in orchestrating osteogenesis.

Previous studies have established a link between Notch signaling and osteogenesis.^[^ ^19^, ^34^, ^35^, ^36^, ^37^ ^]^ Consistent with these findings, inhibition of Notch signaling in our co‐culture model, through treatment with DAPT, significantly impaired the expression of early osteogenic markers in bm‐MSCs (Figure 7C). This result confirms the critical role of Notch signaling in the osteogenic differentiation of bm‐MSCs, as promoted by both b‐ECs and c‐Kit+ HPCs. To validate the efficacy of DAPT treatment, we performed a series of control experiments. Dose–response titration experiments confirmed that 10 µm DAPT was sufficient to achieve robust suppression of canonical Notch target genes *HEY1* and *HEY2*, as well as osteogenic markers *SP7 (OSX)* and *RUNX2*, with no additional effect at higher concentrations (Figure S8A,B, Supporting Information). Western blot analyses further demonstrated that DAPT treatment effectively reduced NOTCH1 protein levels in b‐ECs, HPCs, and bm‐MSCs (Figure S8C, Supporting Information). In addition, DAPT‐treated bm‐MSCs isolated from 3D co‐cultures exhibited decreased protein levels of the osteogenic marker osteopontin (OPN), corroborating impaired osteogenic differentiation at the protein level (Figure S8D, Supporting Information). These results confirm the specificity and efficacy of Notch pathway inhibition under the experimental conditions used.

To further explore the collaborative roles of b‐ECs and c‐Kit+ HPCs in osteogenesis, we devised a two‐stage in vitro model. Initially, c‐Kit+ HPCs were co‐cultured with b‐ECs to simulate the KITLG‐dependent recruitment and activation phase (Stage 1, S1; Figure 7D). Subsequently, these activated c‐Kit+ HPCs—isolated as CD31‐ cells (denoted as *HPCs in Figure 7)—were introduced to bm‐MSCs within our established 3D hydrogel matrix model (Stage 2, S2; Figure 7D). Analysis of osteogenic markers in bm‐MSCs isolated from S2 revealed that *HPCs alone significantly enhanced the osteogenic differentiation of bm‐MSCs compared to their non‐activated HPC counterparts (Figure 7E). Furthermore, using this two‐stage model, we confirmed that disruption of Notch signaling at either stage diminished the osteogenic potential of bm‐MSCs (Figure 7F), underscoring the central role of Notch signaling in both b‐EC‐mediated HPC activation and the subsequent osteogenic induction by activated *HPCs. Consistent with this dual role, selective inhibition of Notch signaling during either Stage 1 or Stage 2 impaired canonical Notch target gene expression in bm‐MSCs, confirming that Notch signaling is essential at both steps of this osteoinductive cascade (Figure S9, Supporting Information).

Together, these results highlight the role of b‐ECs and c‐Kit+ HPCs in driving bm‐MSC osteogenesis, emphasizing the contributions of KITLG and Notch signaling pathways in this process. Specifically, our findings demonstrate that Notch signaling is required at two distinct stages: first, during the activation of HPCs by b‐ECs, and second, during the subsequent induction of osteogenic differentiation in bm‐MSCs by the activated HPCs.

## 3 Discussion

**Figure 8:**
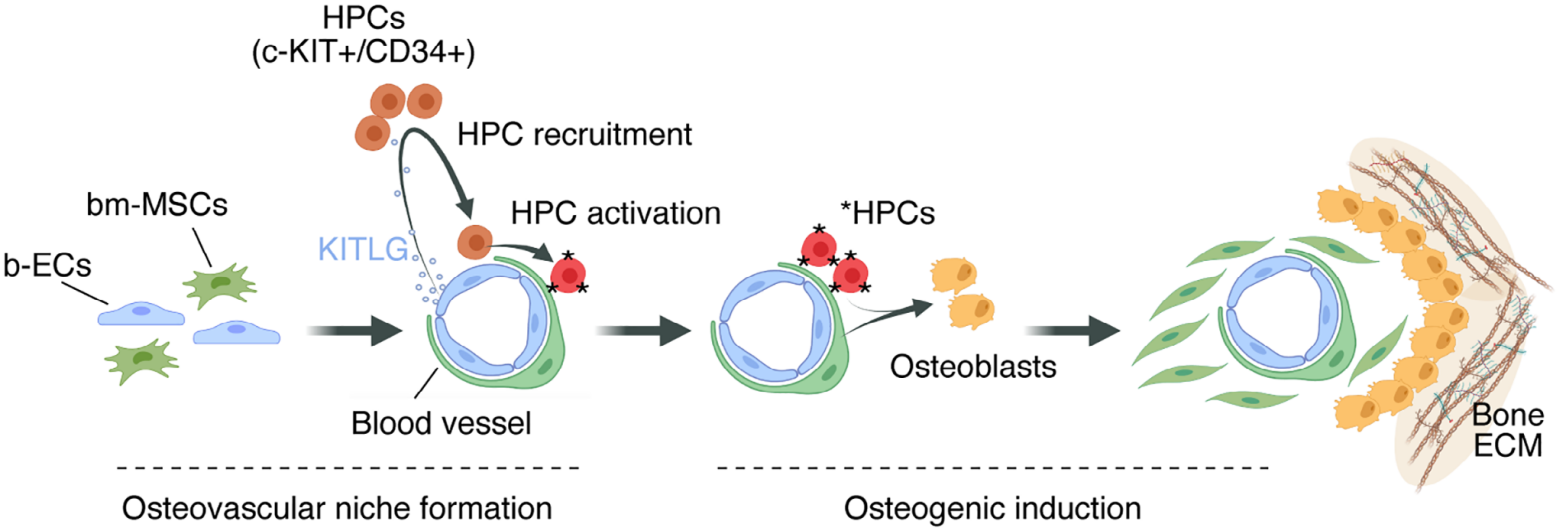
Osteogenesis mediated by b‐ECs through KITLG expression and HPC recruitment. Schematic illustrating the osteoinductive mechanism of bone‐derived b‐ECs via KITLG signaling. b‐ECs uniquely express KITLG, which recruits c‐Kit+/CD34+ HPCs to the osteovascular niche, initiating a cascade that culminates in the osteogenic differentiation of bm‐MSCs and subsequent ossification. This process underscores the potential of b‐ECs to enhance bone regeneration and repair by establishing an osteoinductive microenvironment. This diagram was partially created with BioRender.com.

In this study, we have revealed that b‐ECs are endowed with unique osteoinductive properties, distinguishing them from other non‐bone‐derived human ECs. These properties enable b‐ECs to promote robust bone formation in vivo. Critically, our findings reveal that this distinct osteoinductive potential is mediated by the expression of KITLG in b‐ECs. KITLG is instrumental in directing the recruitment of c‐Kit+/CD34+ HPCs to the osteovascular niche, thereby initiating a sequence of events that leads to robust osteogenic differentiation of bm‐MSCs and, in turn, tissue mineralization and bone formation (**Figure** **8**). These findings not only deepen our understanding of the intricate cellular interplay of the vascular‐bone interface but also open new therapeutic possibilities for employing b‐ECs in bone regeneration and repair.

The organotypic specificity and paracrine role of ECs are increasingly important areas of study in vascular biology. Tissue‐specific ECs establish specialized vascular niches, deploying growth factors known as angiocrine factors.^[^ ^38^, ^39^ ^]^ Research has shown that these angiocrine factors are active participants in organ development, regeneration, homeostasis, and metabolism.^[^ ^39^, ^40^, ^41^, ^42^ ^]^ For example, ECs in the lungs, liver, and neural tissues release unique angiocrine factors that stimulate stem cell renewal and expansion, aiding in tissue regeneration after injury.^[^ ^43^, ^44^, ^45^ ^]^ This specialization of ECs confers them with distinct phenotypic, structural, functional, and secretory attributes, creating specialized vascular subpopulations with organotypic and disease‐specific signatures.

Our research contributes to this understanding by demonstrating that human b‐ECs also exhibit distinct osteoinductive properties not found in ECs from other human tissues. This is mediated by the secretion of the angiocrine factor KITLG, which orchestrates the differentiation of bm‐MSCs into the osteogenic lineage. This mechanism sheds light on how b‐ECs can influence bone‐specific stem cell differentiation. Furthermore, an important aspect of our investigation is the demonstration that human b‐ECs maintain their osteoinductive properties even after isolation and culture. This challenges the previously held belief that tissue‐specific endothelial signatures are dependent on the in vivo microenvironment. Our findings suggest that b‐ECs retain a degree of their organotypic identity ex vivo, opening new possibilities for using these cells in therapeutic applications for bone repair.

The relationship between vasculature and osteogenesis, particularly the paracrine role of the endothelium, has been increasingly recognized, though primarily in developmental contexts and murine models. Pioneering work by Ralf Adams and colleagues in mice has shown that bone endothelium‐secreted factors critically regulate the osteogenic activity of osteoprogenitor cells or bm‐MSCs.^[^ ^19^, ^20^ ^]^ These studies highlighted a unique subset of ECs in long bones, characterized by strong CD31 and Endomucin (Emcn) staining, a feature distinct from the rest of the bone vasculature. This led to the classification of bone microvessels into two types: the less common CD31^hi^/Emcn^hi^ type H vessels and the more prevalent CD31^lo^/Emcn^lo^ type L sinusoidal vessels, each with distinct expression profiles and functional properties. Notably, type H endothelium was found to mediate the osteogenic differentiation of bm‐MSCs.

Our study extends these insights to the human context, demonstrating that human b‐ECs possess distinct osteoinductive properties not found in other ECs. This observation is aligned with the murine findings and suggests that such properties are retained postnatally, not limited to developmental stages. Our b‐ECs, primarily arteriolar rather than sinusoidal, may parallel the murine‐type H vessels, though further studies are needed to establish their equivalence. This finding contributes to the expanding understanding of EC diversity and their specific roles in bone biology, indicating that human b‐ECs could be integral to bone regeneration strategies beyond the developmental context.

Our research also aligns with previous murine developmental studies indicating the essential role of Notch signaling in the link between ECs and osteogenesis.^[^ ^19^, ^46^ ^]^ These studies found that Notch signaling, contrary to its known function in other organs and tumors,^[^ ^47^, ^48^ ^]^ promotes EC proliferation and vessel growth in postnatal long bones. Disruption of Notch signaling in these studies led to impaired bone vessel morphology, reduced osteogenesis, and decreased bone mass, highlighting the role of angiocrine signals like Noggin, which is regulated by Notch. Our study complements these findings, as we observed that inhibition of Notch signaling impaired the osteogenic differentiation of bm‐MSCs influenced by human b‐ECs. This parallel suggests the potential significance of Notch signaling in human EC‐mediated bone regeneration, offering a promising avenue for future therapeutic interventions.

The expression of KITLG in b‐ECs as an angiocrine factor mediating osteogenesis is a key discovery of our study. Prior studies have confirmed the expression of KITLG in the bone vasculature, underlining its crucial role in the bone marrow microenvironment.^[^ ^49^, ^50^, ^51^ ^]^ Furthermore, the identification of a distinct subtype of bone marrow ECs in the epiphysis that expresses KITLG suggests an important spatial heterogeneity within the bone vasculature.^[^ ^50^ ^]^ Additionally, the presence of KITLG and Notch ligands (Dll4, Dll1) in bone vascular cells has been established, highlighting the intricate interactions within this environment.^[^ ^51^ ^]^ These insights significantly contribute to our understanding of the molecular characteristics and diversity of bone vascular cells.

In our studies, the expression of KITLG in culture‐expanded b‐ECs is consistent with previous findings in murine models, where KITLG was predominantly expressed in bone arteriole ECs, unlike in sinusoidal ECs.^[^ ^28^ ^]^ However, in these earlier studies, KITLG's primary recognition was its vital role in sustaining hematopoietic niches, particularly through its binding to the c‐Kit receptor on hematopoietic stem cells (HSCs). This interaction is essential for fostering an environment conducive to HSC survival, proliferation, and differentiation, particularly under stress or during regeneration.^[^ ^27^, ^28^, ^29^, ^30^ ^]^ Additional studies indicated an association between the deletion of membrane‐bound KITLG and disruptions in c‐Kit signaling with alterations in bone mass and turnover.^[^ ^52^, ^53^ ^]^ However, these studies did not delineate the osteogenic influence through the lens of b‐EC‐specific KITLG expression. Our research bridges this gap by demonstrating, via loss‐of‐function and gain‐of‐function studies, that KITLG expression is both necessary and sufficient for endowing ECs with osteoinductive properties. This finding positions KITLG as a central target in developing therapeutic strategies for stimulating osteogenesis and enhancing bone regeneration.

Our investigations into the mechanisms by which KITLG expression in b‐ECs contributes to osteogenesis have revealed a crucial involvement of bone marrow‐derived c‐Kit+/CD34+ HPCs. We established b‐ECs as the central orchestrator in this process, with KITLG directing the recruitment of HPCs to the osteovascular niche, thereby initiating a cascade leading to the osteogenic differentiation of bm‐MSCs and subsequent bone formation. Our study aligns with prior research highlighting c‐Kit+/CD34+ HPCs' involvement in osteogenesis. For instance, these progenitor cells have been shown to be mobilized for bone healing after fractures,^[^ ^54^, ^55^ ^]^ and to reverse osteoporosis by influencing osteoblastic and osteoclastic functions in mouse models.^[^ ^56^ ^]^ Our research advances this understanding by demonstrating that b‐ECs, through KITLG expression, not only recruit but also activate HPCs. These activated HPCs* are self‐sufficient in initiating the osteogenic differentiation of bm‐MSCs. Hence, our data indicates an indirect mediation of osteogenesis by b‐ECs through the sequential recruitment and activation of c‐Kit+ HPCs, processes regulated by Notch signaling.

Our study establishes a mechanistic framework by which b‐ECs mediate osteogenesis: KITLG expression by b‐ECs recruits c‐Kit+ HPCs, activates them, and primes a Notch‐dependent signaling cascade that culminates in bm‐MSC osteogenic differentiation. Using a two‐stage in vitro model, we showed that Notch signaling is required both during HPC activation by b‐ECs and during the subsequent HPC–bm‐MSC interaction, with inhibition at either stage impairing osteogenic outcomes. These findings define Notch as a central mediator linking vascular recruitment signals to osteogenic differentiation, providing new insight into how vascular niches regulate bone formation.

It is important to note that most previous studies have suggested that CD34+ HPCs may contribute to bone regeneration through either direct differentiation into osteoblasts or through paracrine actions, such as VEGF secretion aiding vascularization and indirectly supporting osteogenesis.^[^ ^57^ ^]^ However, our study adds a novel aspect to these possible mechanisms by demonstrating a direct interaction between bm‐MSCs and HPCs. This insight further contributes to the recognized therapeutic potential of HPCs, corroborated in the context of bone regeneration by large animal model studies ^[^ ^58^ ^]^ and later pursued in early‐phase clinical trials.^[^ ^59^ ^]^

Acknowledging the limitations of our study is important for contextualizing its findings. One limitation is the use of a cranial orthotopic model, which involves low‐weight‐bearing bone and may not fully represent the regenerative demands of higher‐stress bones. While this model is not intended to evaluate weight‐bearing bone regeneration, it is well‐established for assessing the regenerative potential of cells in non‐load‐bearing bones, such as the calvarium. This makes it a valuable platform for demonstrating the unique advantages of b‐ECs in bone regeneration. However, the effectiveness of b‐ECs in inducing bone regeneration in mechanically demanding environments remains to be explored.

Another consideration is the age of the donors from whom the b‐ECs were isolated. Our study utilized cells from juvenile‐adolescent donors, leaving the potential of b‐ECs from adult and diseased donors unexplored. This raises the question of whether the osteoinductive properties of b‐ECs vary with age or under pathological conditions. Additionally, the extent to which b‐ECs retain their osteoinductive properties ex vivo is not fully understood. In our study, b‐ECs were cultured for a limited number of passages to preserve their in vivo phenotype, but prolonged culture could potentially alter their regenerative capabilities, which warrants further investigation.

Lastly, the use of xenograft models with immunodeficient mice is another limitation of our study. While these models were essential for studying the regenerative potential of human cells without the complication of immune rejection, they do not fully replicate the environment of an immunocompetent organism. Future studies should explore the potential of b‐ECs in bone regeneration within immunocompetent syngeneic animal models or murine humanized models to provide a more comprehensive understanding of these processes in a fully functional immune environment.

Despite these limitations, our study presents promising opportunities for clinical translation. First, our findings suggest the feasibility of isolating b‐ECs from trabecular bone samples, which are commonly obtained during bone autografts. These b‐ECs, with their retained osteoinductive capabilities, could be combined with bm‐MSCs in future therapies for effective bone regeneration. This approach offers a direct path for clinical application, utilizing routine surgical procedures to source b‐ECs for therapeutic use.

Second, the use of osteoinductive b‐ECs could complement existing efforts in bone tissue engineering, which have demonstrated efficacy through the use of pro‐angiogenic and pro‐osteogenic growth factors, with or without cells, in various types of scaffolds.^[^ ^60^, ^61^, ^62^, ^63^, ^64^, ^65^, ^66^ ^]^ A key translational advantage of using b‐ECs is their ability to induce bm‐MSC differentiation without the need for exogenous osteoinductive factors, such as BMP‐2 or parathyroid hormones, which have been associated with undesirable effects, including heterotopic bone formation, raising concerns about their clinical applicability.^[^ ^16^, ^17^, ^18^ ^]^

Lastly, our study underscores the importance of EC specificity in bone regeneration. While obtaining b‐ECs from patients might introduce morbidity and logistical challenges for clinical translation, our research suggests an intriguing alternative by inducing KITLG expression in more readily accessible ECs. This approach could involve using circulating endothelial progenitors, such as ECFCs, or endothelial cells derived from induced pluripotent stem cells (iPSCs), both of which offer less invasive and more translational options. The clinical potential of these strategies, particularly through activating KITLG expression, warrants further investigation in the context of bone regeneration therapies.

## 4 Experimental Section

### Cell Isolation and Culture

De‐identified human iliac crest trabecular bone samples were obtained from discarded surgical material under an Institutional Review Board (IRB)‐approved protocol at Boston Children's Hospital (Protocol Number: NS09‐07‐0377). As these samples were excess clinical material, no patient consent was required. Human bone endothelial cells (b‐ECs) were isolated by enzymatic digestion using Dispase (2.5 µ mL^−1^), Collagenase ll and A (0.25 mg mL^−1^ each), Calcium Chloride Dihydrate (126 µm), and Magnesium Sulfate Heptahydrate (80 µm) in DMEM media. CD31+ cells were then selected using Dynabeads (Invitrogen). The b‐ECs were cultured on 1% gelatin‐coated plates in endothelial cell medium (PromoCell) supplemented with TGFβ inhibitor (10 µm; SB431542, Selleckchem).

Human hematopoietic progenitor cells (HPCs) were isolated from the same bone samples via similar enzymatic digestion and magnetic‐activated cell sorting (MACS) of Lin‐, CD34+ cells (Miltenyi Biotec). HPCs were cultured in suspension plates using StemMACS HSC Expansion Media XF and StemMACS HSC Expansion Cocktail (Miltenyi Biotec).

Human endothelial colony‐forming cells (ECFCs) from umbilical cord blood and human white adipose tissue endothelial cells (wat‐ECs) from subcutaneous WAT samples were isolated in accordance with Institutional Review Board‐approved protocols as previously described.^[^ ^67^ ^]^ ECFCs and wat‐ECs were purified using CD31‐coated magnetic beads (Dynabeads, Thermo Fisher) and cultured in endothelial cell medium on 1% gelatin‐coated plates.

Human bone marrow‐derived mesenchymal stem cells (bm‐MSCs) were isolated and cultured as previously described,^[^ ^13^ ^]^ using MSC‐medium consisting of MSCGM (Lonza), 10% fetal bovine serum, and 1× glutamine–penicillin–streptomycin on uncoated plates.

### Cell Seeding in Biphasic Scaffolds for Implantation

Cells were centrifuged to form a pellet and resuspended in ice‐cold, phenol red‐free Matrigel (BD Bioscience) at 4 °C. For seeding combinations of bm‐MSCs and ECFCs, the ratio was 2:3 ECFCs to bm‐MSCs, using a total of 2 × 10^6^ cells per construct. When combining bm‐MSCs with ECFCs and b‐ECs, ECFCs and b‐ECs were mixed in a 1:1 ratio. Constructs with only bm‐MSCs utilized 1.2 × 10^6^ cells. The cell‐Matrigel mixtures, totaling 50 µL each, were applied to the biphasic scaffold consisting of a chondroitin sulfate (CS)‐cryogel containing whitlockite nanoparticles, synthesized as previously described.^[^ ^21^ ^]^ The scaffolds were fabricated using a cylindrical mold (4 mm in diameter and 1 mm in height) to ensure uniformity. Gelation was completed by a 10‐min incubation at 37 °C, preparing the scaffolds for subcutaneous implantation.

### Ectopic Subcutaneous Implantation of Scaffolds

All animal procedures were performed following protocols approved by the Institutional Animal Care and Use Committee (IACUC) at Boston Children's Hospital (Protocol Number: 0 0 002065). Athymic nude (nu/nu) mice, aged 5 to 6 weeks, were obtained from Envigo RMS, Inc. Mice were housed in compliance with Boston Children's Hospital guidelines. Scaffolds seeded either with bm‐MSCs alone or in combination with ECs were implanted subcutaneously for up to 8 weeks. Each time point involved a minimum of three mice to ensure independent replication of experiments. Surgical implantation involved minimal skin incisions in the subcutaneous space on the backs of the mice, performed under aseptic conditions and anesthesia.

### Orthotopic Calvarial Implantation of Scaffolds

Critical‐sized calvarial bone defects were surgically generated on adults (8 weeks old) nude mice.^[^ ^24^ ^]^ A midline incision was made on the scalp to expose the right parietal bone, followed by blunt scraping to remove the pericranium. Subsequently, a unilateral 4‐mm full‐thickness defect was created in the right non‐suture associated parietal bone using a sterile diamond‐coated trephine drill bit. Grafts containing bm‐MSCs with either ECFCs or a combination of ECFCs and b‐ECs were then implanted into the calvarial defects. These grafts were maintained for a period ranging from 1 to 8 weeks, after which a terminal procedure was conducted to harvest the grafts for further analysis.

### Mechanical Properties Assessment

Scaffolds were explanted at designated intervals (Weeks 1, 4, 6, and 8) for mechanical characterization. Their stress‐strain profiles were determined using a universal mechanical testing apparatus (EZ‐Test, Shimadzu) with a 10 kN load cell. The compressive modulus (Young's modulus) was calculated from the linear portion of these stress‐strain curves.

### Nanoindentation Analysis

The elastic modulus and contact hardness of the scaffolds were evaluated using nanoindentation, as previously described.^[^ ^21^ ^]^ Scaffolds underwent fixation in 4% paraformaldehyde for a day, followed by embedding in Ortho‐Jet acrylic resin. The embedded samples were sectioned into 2 mm slices with an Isomet low‐speed diamond saw. The slices were polished using silicon carbide paper and aluminum oxide paste, then attached to a stainless‐steel holder on a Nano‐XP nanoindenter (MTS). Indentations, up to 500 nm depth with loading and unloading rates of 10 nm s^−1^, were made. Force‐displacement curves from these indentations provided data for calculating contact hardness and elastic modulus, ensuring a 30 µm separation between indentations to avoid influence from adjacent points.

### Micro‐Computed Tomography Analysis

Micro‐computed tomography (µCT) was used for quantifying bone formation within scaffolds as previously described.^[^ ^21^ ^]^ The scans were performed using a SkyScan 1272 (Bruker) at settings of 59 kV and 167 µA with a 40 ms exposure time without a filter. X‐ray projections were collected at 0.6° intervals over a 360° rotation. An automated threshold algorithm segmented the datasets. The projected images were reconstructed into 3D images using NRecon Micro‐CT software provided by Bruker.

### Overexpression and Silencing of KITLG

To overexpress KITLG in ECs, gateway cloning was performed for the insertion of KITLG gateway entry clone (GC‐OG01008, GeneCopoeia) into the pLEX_305 vector (Plasmid #41 390, Addgene), followed by generating lentiviral particles using 293T cells and the infection of ECs. Puromycin selection was performed to generate the stable cell line expressing the pLEX305‐KITLG plasmid, as validated by qPCR and ELISA in conditioned media.

The silencing of KITLG in ECs was achieved utilizing the *KITLG* shRNA lentiviral particles (Santa Cruz Biotechnology) according to the manufacturer's instructions. Briefly, *KITLG* shRNA lentiviral particles were mixed with Polybrene (Santa Cruz Biotechnology) at 10 µg mL^−1^ in the media that was added to ECs seeded on the previous day to achieve a 50% confluency. The media containing the lentiviral particles and Polybrene was removed the next day and replaced by fresh growth media. Puromycin selection was performed to generate the stable cell line with KITLG silencing, as validated by qPCR and ELISA in conditioned media.

### In Vitro Osteogenesis Model

To model osteogenesis in vitro, bm‐MSCs (2.5 × 10^4^ cells) were cultured either alone or in combination with b‐ECs (2.5 × 10^5^) and/or c‐kit+ HPCs (1.5 × 10^4^) within a 3D collagen/fibrin hydrogel scaffold (100 µL) for 7 days. This hydrogel was formulated using 1.5 mg mL^−1^ cultrex bovine collagen I (R&D Systems), 30 µg mL^−1^ human fibronectin (Millipore Sigma), 10% v/v FBS, 25 mm HEPES, 3 mg mL^−1^ fibrinogen (Sigma‐Aldrich), 3% H2O, and 10% Medium 199 with Earle's salts (10X, ThermoFisher Scientific). For bm‐MSC isolation at the end of the culture period, the 3D gel was enzymatically digested (2.5 u mL^−1^ dispase, 0.25 mg mL^−1^ collagenase II, 0.25 mg mL^−1^ collagenase A, 126 µm calcium chloride dihydrate, 80 µm magnesium sulfate heptahydrate in DMEM media). bm‐MSCs were then separated using a PE anti‐human CD90 antibody (BioLegend) and magnetic‐activated cell sorting with anti‐PE microbeads (Miltenyi Biotec).

For experiments involving Notch signaling inhibition, DAPT (N‐[N‐(3,5‐difluorophenacetyl)‐L‐alanyl]‐S‐phenylglycine t‐butyl ester; Sigma‐Aldrich) was added to the culture medium at a final concentration of 10 µm. This dose was selected based on dose–response titration experiments, which demonstrated that 10 µm DAPT was sufficient to achieve robust suppression of canonical Notch target genes (*HEY1*, *HEY2*) (Figure S8A,B, Supporting Information). In the two‐stage in vitro model, DAPT was applied either during the initial co‐culture of b‐ECs and HPCs (Stage 1) or during the subsequent co‐culture of activated *HPCs with bm‐MSCs (Stage 2), depending on the experimental design.

For the two‐stage in vitro osteogenesis model, the HPC activation stage (Stage 1) was achieved by co‐culture of c‐Kit+ HPCs with b‐ECs for 3 days. For Stage 2, the activated c‐Kit+ HPCs (*HPCs) were isolated as CD31‐ cells using Dynabeads (Invitrogen) and then introduced to bm‐MSCs within the established 3D hydrogel matrix model as described above. After 7 days of osteogenic induction, bm‐MSCs were isolated for subsequent qPCR analysis using a PE anti‐human CD90 antibody (BioLegend) and magnetic‐activated cell sorting with anti‐PE microbeads (Miltenyi Biotec).

### Quantitative Real‐Time PCR

Unless otherwise specified, total RNA was extracted from cells using an RNeasy mini kit (Qiagen). For the in vitro osteogenesis 3D gel modeling system, total RNA was extracted using the Power SYBR Green Cells‐to‐CT kit (Invitrogen). RNA concentration was quantified using a Nanodrop (Thermo Fisher), and RNA purity was assessed by measuring the absorbance ratio at 260 and 280 nm. Subsequently, cDNA synthesis was carried out using either the high‐capacity cDNA reverse transcription kit (Thermo Fisher) or the Power SYBR™ Green Cells‐to‐CT kit. Quantitative real‐time PCR assays were performed using SYBR Green MasterMix (Thermo Fisher) or Power SYBR Green MasterMix (Invitrogen), with GAPDH as the reference gene. Primer sequences utilized for real‐time PCR are available in Table S1 (Supporting Information).

### Histological Analysis

Explant scaffolds were fixed overnight in 10% buffered formalin, embedded in paraffin, and sectioned into 7 µm‐thick slices. Deparaffinized and hydrated slides were stained with H&E to visualize microvessels. Microvessel density was quantified as erythrocyte‐filled vessels mm^−2^. Movat pentachrome‐stained sections were employed to detect collagen (yellow), glycosaminoglycans (green), muscle (red), mucin (blue), and fibrin (bright red), assessing collagen deposition and chondroitin sulfate (CS) degradation within scaffolds. For von Kossa staining, slides were incubated in a 5% silver nitrate solution for 60 min with UV light exposure, followed by a 2 min incubation in a 5% sodium thiosulfate solution. Von Kossa‐stained sections were used to quantify tissue mineralization as a percentage of the section area mineralized using image color summarizer (v0.76, Martin Krzywinski).

### Immunostaining Analysis

For immunohistochemical staining, sections underwent deparaffinization, followed by antigen retrieval using a tris–EDTA buffer (pH 9.0) containing 10 mm Tris‐Base, 2 mm EDTA, and 0.05% Tween‐20. Sections were blocked for 30 min with 5% blocking serum and then incubated overnight at 4 °C with primary antibodies. The list of primary and secondary antibodies can be found in Table S2 (Supporting Information). Human ECs were visualized using an anti‐human‐specific CD31 antibody (Agilent), a horseradish peroxidase‐conjugated mouse secondary antibody (1:200, Vector Laboratories), and 3,3′‐diaminobenzidine. Hematoxylin was used for counterstaining, and sections were mounted with Permount (Thermo Fisher). Immunofluorescent staining utilized secondary antibodies labeled with either Texas Red or FITC, followed by DAPI counterstaining. In select experiments, rhodamine‐conjugated Ulex europaeus agglutinin I (UEA‐1) lectin (1:200, Vector Laboratories) was employed for additional fluorescent staining.

### Enzyme‐Linked Immunosorbent Assay (ELISA)

The ELISA for human KITLG followed the manufacturer's instructions for the Human SCF Quantikine ELISA Kit (R&D, DCK00). Briefly, conditioned media was collected after 72 hours of cell culture, centrifuged at 290 g, and the supernatant was collected. In the ELISA microplate, 100 µL of Assay Diluent was added to each well, followed by 100 µL of Standard, control, or sample. The plate was covered with a sealer and incubated at room temperature for 2 h. Afterward, each well underwent three washes. Two hundred microliters of Conjugate were added to each well, covered with a new sealer, and incubated for 2 h at room temperature. The wells were washed three times before the addition of 200 µL Substrate Solution to each well. The microplate was then incubated at room temperature while protected from light. After a 20‐min incubation, 50 µL of Stop Solution was added to each well, and the results were read at 450 nm within 30 min.

### Flow Cytometry

Single‐cell suspensions were prepared and resuspended in phosphate‐buffered saline (PBS) with 2% fetal bovine serum (FBS). To block nonspecific antibody binding, cells were incubated with 20 µg mL^−1^ rat IgG (Sigma‐Aldrich) for 15 min. Subsequently, cells were incubated with either pre‐conjugated or primary antibodies (Table S2, Supporting Information) for 30 min on ice. When secondary antibodies were required, cells were washed with PBS with 2% FBS before incubation with secondary antibodies at 4 °C for 20 min. Stained cells underwent a final wash with PBS with 2% FBS before analysis using a BD Accuri C6 Plus Flow Cytometer System (BD Biosciences). FlowJo software (TreeStar) was employed to analyze the data and generate flow cytometry plots.

To assess blood‐forming ability, ECs and bm‐MSCs (EC:MSC ratio = 2:3; a total of 2 × 10^6^ cells per implant) were suspended in 200 µL of ice‐cold phenol red‐free Matrigel (BD Bioscience) and injected subcutaneously into the flank of athymic nude mice using a 26‐gauge needle, as previously described. Implants were harvested after 1 week for histological evaluation.

### Hematopoietic Colony Forming Unit Assay

The colony‐forming unit (CFU) assay involved plating human bone marrow‐derived HPCs (bm‐HPCs) in methylcellulose and culturing them with methylcellulose complete media following the manufacturer's instructions (R&D Systems). Human cord blood‐derived HPCs were used as a control. After 14 days, the number of colonies produced by different CFU subtypes was counted using an inverted microscope. Colonies were identified based on cell morphology, and for erythroid and mixed colonies, the presence of red cells expressing hemoglobin. Colonies were categorized as erythroid (BFU‐E, CFU‐E with BM), myeloid (CFU‐G/M/GM), and mixed (CFU‐GEMM).

### Tube Formation Assay

To assess the angiogenic ability of endothelial cells (ECs), a tube formation assay was performed. Briefly, 100 µL Matrigel (BD Bioscience) was added to each well of a 48‐well plate and allowed to gelation at 37 °C for 30 min. ECs were then seeded onto the solidified Matrigel at a density of 10 000 cells cm^−2^ and incubated under standard culture conditions to allow network formation for 12 h. Phase‐contrast microscopy was used to capture images of the formed tubular structures. Quantitative analysis of the vascular network was conducted using the AngioTool 2.0 plugin in ImageJ.

### Bulk RNA Sequencing

For RNA‐Seq analysis, total RNA from cultured‐expanded b‐ECs, wat‐ECs, and ECFCs was extracted using the RNeasy Mini Kit (Qiagen). Library construction and sequencing were performed at the Integrated Genomics Operation (IGO) core at Massachusetts General Hospital (MGH). Sequencing utilized an Illumina HiSeq2500 platform with a 2 × 150 paired‐end configuration. Quality checks of FASTQ files and reads alignment to the UCSC hg38 genome were done using FastQC, Qualimap, and STAR, respectively. Transcript expression was quantified against the Ensembl GRCh38 transcriptome using Salmon. DESeq2 was employed for differential gene expression analysis, with significant genes identified based on an adjusted p‐value < 0.05. Principal component analysis (PCA) utilized DESeq2's “plotPCA” function of top differentially expressed genes. Gene ontology analysis of growth factor activity‐related genes (GO:0 0 08083; 162 genes) was conducted using GeneSCF. Counts per million (CPM) data of b‐ECs, wat‐ECs, and ECFCs RNA‐seq results are available in Dataset S1 (Supporting Information).

### Western Blot Analysis

Whole‐cell lysates were extracted using RIPA buffer (Millipore Sigma, R278). Protein concentrations were determined via the bicinchoninic acid (BCA) assay (Thermo Fisher Scientific, 23 227). Equivalent protein amounts from each sample were separated on 4–12% Novex Tris‐Glycine gels (Thermo Fisher Scientific) and transferred to polyvinylidene fluoride (PVDF) membranes (Bio‐Rad) using the Novex Mini‐Cell XCell SureLock system (Invitrogen). Membranes were blocked for 1 h at room temperature in TBST containing 5% non‐fat milk, then incubated overnight at 4 °C with primary antibodies, respectively, as listed in Table S2 (Supporting Information). After washing, membranes were incubated with HRP‐conjugated secondary antibodies. Protein signals were detected using an enhanced chemiluminescence (ECL) substrate (Kindle Biosciences, Western Blot Detection Kit, R1004).

### Microscopy Imaging

Imaging was conducted using an Axio Observer Z1 inverted microscope (Carl Zeiss), with image processing via AxioVision Rel. 4.8 software. Fluorescent images were captured using a 20x objective lens. Non‐fluorescent images were obtained with an AxioCam MRc5 camera, employing 5x and 20x objective lenses.

### Statistics and Reproducibility

All experiments were independently repeated at least three times with similar results. Specifically, all micrographs presented in the figures were representative of experiments conducted on three or more independent occasions. Except where specifically mentioned, data were presented as means ± standard error of the mean (s.e.m.). When comparing two groups, mean values were compared using unpaired two‐tailed Student's t‐tests. Multiple group comparisons were conducted through analysis of variance (one‐way ANOVA) followed by Bonferroni correction. No exclusion criteria were applied to any of the analyses. All statistical calculations were performed using GraphPad Prism v.9 software (GraphPad Software Inc.). Statistical significance was set at *p* < 0.05.

## Conflict of Interest

The authors declare no conflict of interest.

## Author Contributions

X.L. and H.D.K. contributed equally to this work. X.L., H.D.K., R.Z.L., and J.M.M. conceived and designed the project. X.L., H.D.K., A.C.L., L.G., Y.Z., C.N.L., X.H., C.S., M.A., Y.H.A., M.J.P., D.G.K., B.L.P., and R.Z.L. performed the experimental work. N.S.W. and J.M.M. acquired funding. A.K.G., N.S.H., and J.M.M. supervised the project. X.L. and J.M.M. wrote the original draft. X.L., H.D.K., A.C.L., L.G., Y.Z., C.N.L., X.H., C.S., M.A., Y.H.A., M.J.P., D.G.K., A.K.G., B.L.P., N.S.W., R.Z.L., and J.M.M. reviewed and edited the manuscript.

## Supporting information



Supporting Information

Supplemental Dataset 1

Supplemental Video 1

## Data Availability

Source data are provided with this article (Dataset S1, Supporting Information).

## References

[1] W. G. D. Long , T. A. Einhorn , K. Koval , M. McKee , W. Smith , R. Sanders , T. Watson , J. Bone Jt. Surg. 2007, 89, 649. 10.2106/JBJS.F.00465 17332116

[2] R. M. Kline , S. A. Wolfe , Plast. Reconstr. Surg. 1995, 95, 5. 7809267

[3] S. W. S. Laurie , L. B. Kaban , J. B. Mulliken , J. E. Murray , Plast. Reconstr. Surg. 1984, 73, 933. 6374708 10.1097/00006534-198406000-00014

[4] A. K. Greene , J. B. Mulliken , M. R. Proctor , G. F. Rogers , Plast. Reconstr. Surg. 2008, 122, 563. 18626375 10.1097/PRS.0b013e31817d61c1

[5] W. J. Koenig , J. M. Donovan , J. M. Pensler , Plast. Reconstr. Surg. 1995, 95, 1. 7809219 10.1097/00006534-199501000-00001

[6] A. Stahl , Y. P. Yang , Tissue Eng. Part B: Rev. 2021, 27, 539. 33138705 10.1089/ten.teb.2020.0281 PMC8739850

[7] P. Bianco , X. Cao , P. S. Frenette , J. J. Mao , P. G. Robey , P. J. Simmons , C.‐Y. Wang , Nat. Med. 2013, 19, 35. 23296015 10.1038/nm.3028 PMC3998103

[8] C. Nombela‐Arrieta , J. Ritz , L. E. Silberstein , Nat. Rev. Mol. Cell Bio 2011, 12, 126. 21253000 10.1038/nrm3049 PMC3346289

[9] M. Crisan , S. Yap , L. Casteilla , C.‐W. Chen , M. Corselli , T. S. Park , G. Andriolo , B. Sun , B. Zheng , L. Zhang , C. Norotte , P.‐N. Teng , J. Traas , R. Schugar , B. M. Deasy , S. Badylak , H.‐J. Bűhring , J.‐P. Giacobino , L. Lazzari , J. Huard , B. Péault , Cell Stem Cell 2008, 3, 301. 18786417 10.1016/j.stem.2008.07.003

[10] A. Greenbaum , Y.‐M. S. Hsu , R. B. Day , L. G. Schuettpelz , M. J. Christopher , J. N. Borgerding , T. Nagasawa , D. C. Link , Nature 2013, 495, 227. 23434756 10.1038/nature11926 PMC3600148

[11] Y. Kunisaki , I. Bruns , C. Scheiermann , J. Ahmed , S. Pinho , D. Zhang , T. Mizoguchi , Q. Wei , D. Lucas , K. Ito , J. C. Mar , A. Bergman , P. S. Frenette , Nature 2013, 502, 637. 24107994 10.1038/nature12612 PMC3821873

[12] B. Sacchetti , A. Funari , S. Michienzi , S. D. Cesare , S. Piersanti , I. Saggio , E. Tagliafico , S. Ferrari , P. G. Robey , M. Riminucci , P. Bianco , Cell 2007, 131, 324. 17956733 10.1016/j.cell.2007.08.025

[13] R.‐Z. Lin , R. Moreno‐Luna , D. Li , S.‐C. Jaminet , A. K. Greene , J. M. Melero‐Martin , Proc. Natl. Acad. Sci. USA 2014, 111, 10137. 24982174 10.1073/pnas.1405388111 PMC4104912

[14] L. Cao , J. Wang , J. Hou , W. Xing , C. Liu , Biomaterials 2014, 35, 684. 24140042 10.1016/j.biomaterials.2013.10.005

[15] D. H. R. Kempen , L. Lu , T. E. Hefferan , L. B. Creemers , A. Maran , K. L. Classic , W. J. A. Dhert , M. J. Yaszemski , Biomaterials 2008, 29, 3245. 18472153 10.1016/j.biomaterials.2008.04.031 PMC2577841

[16] G. S. Krishnakumar , A. Roffi , D. Reale , E. Kon , G. Filardo , Int. Orthop. 2017, 41, 1073. 28424852 10.1007/s00264-017-3471-9

[17] A. W. James , G. LaChaud , J. Shen , G. Asatrian , V. Nguyen , X. Zhang , K. Ting , C. Soo , Tissue Eng. Part B Rev. 2016, 22, 284. 26857241 10.1089/ten.teb.2015.0357 PMC4964756

[18] A. C. Carreira , F. H. Lojudice , E. Halcsik , R. D. Navarro , M. C. Sogayar , J. M. Granjeiro , J. Dent. Res. 2013, 93, 335. 10.1177/0022034513518561 24389809

[19] S. K. Ramasamy , A. P. Kusumbe , L. Wang , R. H. Adams , Nature 2014, 507, 376. 24647000 10.1038/nature13146 PMC4943529

[20] A. P. Kusumbe , S. K. Ramasamy , R. H. Adams , Nature 2014, 507, 323. 24646994 10.1038/nature13145 PMC4943525

[21] H. D. Kim , X. Hong , Y. An , M. J. Park , D. Kim , A. K. Greene , B. L. Padwa , N. S. Hwang , R. Lin , J. M. Melero‐Martin , Adv. Healthcare Mater. 2021, 10, 2100070. 10.1002/adhm.202100070 PMC8273143 33882194

[22] H. D. Kim , H. L. Jang , H.‐Y. Ahn , H. K. Lee , J. Park , E. Lee , E. A. Lee , Y.‐H. Jeong , D.‐G. Kim , K. T. Nam , N. S. Hwang , Biomaterials 2017, 112, 31. 27744219 10.1016/j.biomaterials.2016.10.009

[23] H. D. Kim , E. A. Lee , Y.‐H. An , S. L. Kim , S. S. Lee , S. J. Yu , H. L. Jang , K. T. Nam , S. G. Im , N. S. Hwang , ACS Appl. Mater. Interfaces 2017, 9, 21639. 28605908 10.1021/acsami.7b04114

[24] D. M. Gupta , M. D. Kwan , B. J. Slater , D. C. Wan , M. T. Longaker , J. Craniofacial Surg. 2008, 19, 192. 10.1097/scs.0b013e31815c93b7 18216688

[25] S. Werner , C. Alzheimer , Cytokine Growth Factor Rev. 2006, 17, 157. 16481210 10.1016/j.cytogfr.2006.01.001

[26] M. Namwanje , C. W. Brown , Cold Spring Harb. Perspect. Biol. 2016, 8, a021881. 27328872 10.1101/cshperspect.a021881 PMC4930927

[27] C. N. Inra , B. O. Zhou , M. Acar , M. M. Murphy , J. Richardson , Z. Zhao , S. J. Morrison , Nature 2015, 527, 466. 26570997 10.1038/nature15530 PMC4838203

[28] C. Xu , X. Gao , Q. Wei , F. Nakahara , S. E. Zimmerman , J. Mar , P. S. Frenette , Nat. Commun. 2018, 9, 2449. 29934585 10.1038/s41467-018-04726-3 PMC6015052

[29] L. Ding , T. L. Saunders , G. Enikolopov , S. J. Morrison , Nature 2012, 481, 457. 22281595 10.1038/nature10783 PMC3270376

[30] S. J. Morrison , D. T. Scadden , Nature 2014, 505, 327. 24429631 10.1038/nature12984 PMC4514480

[31] L. Rönnstrand , Cell Mol. Life Sci. 2004, 61, 2535. 15526160 10.1007/s00018-004-4189-6 PMC11924424

[32] R. Möhle , R. Haas , W. Hunstein , J. Hematother. 1993, 2, 483. 7522108 10.1089/scd.1.1993.2.483

[33] M. Ogawa , Y. Matsuzaki , S. Nishikawa , S. Hayashi , T. Kunisada , T. Sudo , T. Kina , H. Nakauchi , S. Nishikawa , J. Exp. Med. 1991, 174, 63. 1711568 10.1084/jem.174.1.63 PMC2118893

[34] Z. Luo , X. Shang , H. Zhang , G. Wang , P. A. Massey , S. R. Barton , C. G. Kevil , Y. Dong , Am. J. Pathol. 2019, 189, 1495. 31345466 10.1016/j.ajpath.2019.05.005 PMC6699068

[35] Y. Muguruma , K. Hozumi , H. Warita , T. Yahata , T. Uno , M. Ito , K. Ando , J. Cell. Physiol. 2017, 232, 2569. 27735989 10.1002/jcp.25647 PMC5485010

[36] Y. Wagley , A. Chesi , P. K. Acevedo , S. Lu , A. D. Wells , M. E. Johnson , S. F. A. Grant , K. D. Hankenson , Stem Cells 2020, 38, 1332. 32535942 10.1002/stem.3245

[37] Y. Xu , B. Shu , Y. Tian , M. Chelly , M. M. Morandi , S. Barton , X. Shang , Y. Dong , J. Cell. Physiol. 2018, 233, 6921. 29693255 10.1002/jcp.26592

[38] S. Rafii , J. M. Butler , B.‐S. Ding , Nature 2016, 529, 316. 26791722 10.1038/nature17040 PMC4878406

[39] D. J. Nolan , M. Ginsberg , E. Israely , B. Palikuqi , M. G. Poulos , D. James , B.‐S. Ding , W. Schachterle , Y. Liu , Z. Rosenwaks , J. M. Butler , J. Xiang , A. Rafii , K. Shido , S. Y. Rabbany , O. Elemento , S. Rafii , Dev. Cell 2013, 26, 204. 23871589 10.1016/j.devcel.2013.06.017 PMC3873200

[40] O. Cleaver , D. A. Melton , Nat. Med. 2003, 9, 661. 12778164 10.1038/nm0603-661

[41] J. Cool , T. J. DeFalco , B. Capel , Proc. Natl. Acad. Sci. USA 2011, 108, 167. 21173261 10.1073/pnas.1010299108 PMC3017142

[42] K. Matsumoto , H. Yoshitomi , J. Rossant , K. S. Zaret , Science 2001, 294, 559. 11577199 10.1126/science.1063889

[43] B.‐S. Ding , D. J. Nolan , P. Guo , A. O. Babazadeh , Z. Cao , Z. Rosenwaks , R. G. Crystal , M. Simons , T. N. Sato , S. Worgall , K. Shido , S. Y. Rabbany , S. Rafii , Cell 2011, 147, 539. 22036563 10.1016/j.cell.2011.10.003 PMC3228268

[44] J. Hu , K. Srivastava , M. Wieland , A. Runge , C. Mogler , E. Besemfelder , D. Terhardt , M. J. Vogel , L. Cao , C. Korn , S. Bartels , M. Thomas , H. G. Augustin , Science 2014, 343, 416. 24458641 10.1126/science.1244880

[45] Q. Shen , S. K. Goderie , L. Jin , N. Karanth , Y. Sun , N. Abramova , P. Vincent , K. Pumiglia , S. Temple , Science 2004, 304, 1338. 15060285 10.1126/science.1095505

[46] C. Xu , V. V. Dinh , K. Kruse , H.‐W. Jeong , E. C. Watson , S. Adams , F. Berkenfeld , M. Stehling , S. J. Rasouli , R. Fan , R. Chen , I. Bedzhov , Q. Chen , K. Kato , M. E. Pitulescu , R. H. Adams , Elife 2022, 11, 60183. 10.7554/eLife.60183 PMC8880996 35119364

[47] R. Benedito , C. Roca , I. Sörensen , S. Adams , A. Gossler , M. Fruttiger , R. H. Adams , Cell 2009, 137, 1124. 19524514 10.1016/j.cell.2009.03.025

[48] I. Noguera‐Troise , C. Daly , N. J. Papadopoulos , S. Coetzee , P. Boland , N. W. Gale , H. C. Lin , G. D. Yancopoulos , G. Thurston , Nature 2006, 444, 1032. 17183313 10.1038/nature05355

[49] Z. Zheng , H. He , X. T. Tang , H. Zhang , F. Gou , H. Yang , J. Cao , S. Shi , Z. Yang , G. Sun , X. Xie , Y. Zeng , A. Wen , Y. Lan , J. Zhou , B. Liu , B. O. Zhou , T. Cheng , H. Cheng , Cell Stem Cell 2022, 29, 1562. 36332570 10.1016/j.stem.2022.10.005

[50] T. Iga , H. Kobayashi , D. Kusumoto , T. Sanosaka , N. Fujita , I. Tai‐Nagara , T. Ando , T. Takahashi , K. Matsuo , K. Hozumi , K. Ito , M. Ema , T. Miyamoto , M. Matsumoto , M. Nakamura , H. Okano , S. Shibata , J. Kohyama , K. K. Kim , K. Takubo , Y. Kubota , Nat. Cell Biol. 2023, 25, 1415. 37798545 10.1038/s41556-023-01240-7 PMC10567563

[51] A. N. Tikhonova , I. Dolgalev , H. Hu , K. K. Sivaraj , E. Hoxha , Á. Cuesta‐Domínguez , S. Pinho , I. Akhmetzyanova , J. Gao , M. Witkowski , M. Guillamot , M. C. Gutkin , Y. Zhang , C. Marier , C. Diefenbach , S. Kousteni , A. Heguy , H. Zhong , D. R. Fooksman , J. M. Butler , A. Economides , P. S. Frenette , R. H. Adams , R. Satija , A. Tsirigos , I. Aifantis , Nature 2019, 569, 222. 30971824 10.1038/s41586-019-1104-8 PMC6607432

[52] S. Lotinun , N. Krishnamra , Sci. Rep. 2016, 6, 31515. 27527615 10.1038/srep31515 PMC4985756

[53] S. Lotinun , G. L. Evans , R. T. Turner , M. J. Oursler , J. Bone Miner. Res. 2005, 20, 644. 15765184 10.1359/JBMR.041209

[54] T. Matsumoto , Y. Mifune , A. Kawamoto , R. Kuroda , T. Shoji , H. Iwasaki , T. Suzuki , A. Oyamada , M. Horii , A. Yokoyama , H. Nishimura , S. Y. Lee , M. Miwa , M. Doita , M. Kurosaka , T. Asahara , J. Cell. Physiol. 2008, 215, 234. 18205179 10.1002/jcp.21309

[55] D. Y. Lee , T.‐J. Cho , J. A. Kim , H. R. Lee , W. J. Yoo , C. Y. Chung , I. H. Choi , Bone 2008, 42, 932. 18326482 10.1016/j.bone.2008.01.007

[56] R. Aggarwal , J. Lu , S. Kanji , M. Joseph , M. Das , G. J. Noble , B. K. McMichael , S. Agarwal , R. T. Hart , Z. Sun , B. S. Lee , T. J. Rosol , R. Jackson , H.‐Q. Mao , V. J. Pompili , H. Das , PLoS One 2012, 7, 39365. 10.1371/journal.pone.0039365 PMC3377665 22724005

[57] T. Matsumoto , A. Kawamoto , R. Kuroda , M. Ishikawa , Y. Mifune , H. Iwasaki , M. Miwa , M. Horii , S. Hayashi , A. Oyamada , H. Nishimura , S. Murasawa , M. Doita , M. Kurosaka , T. Asahara , Am. J. Pathol. 2006, 169, 1440. 17003498 10.2353/ajpath.2006.060064 PMC1698844

[58] N. Rozen , T. Bick , A. Bajayo , B. Shamian , M. Schrift‐Tzadok , Y. Gabet , A. Yayon , I. Bab , M. Soudry , D. Lewinson , Bone 2009, 45, 918. 19665064 10.1016/j.bone.2009.07.085

[59] R. Kuroda , T. Matsumoto , T. Niikura , Y. Kawakami , T. Fukui , S. Y. Lee , Y. Mifune , S. Kawamata , M. Fukushima , T. Asahara , A. Kawamoto , M. Kurosaka , Stem Cells Transl. Med. 2014, 3, 128. 24307697 10.5966/sctm.2013-0106 PMC3902290

[60] C. A. Simmons , E. Alsberg , S. Hsiong , W. J. Kim , D. J. Mooney , Bone 2004, 35, 562. 15268909 10.1016/j.bone.2004.02.027

[61] Y. Huang , D. Kaigler , K. G. Rice , P. H. Krebsbach , D. J. Mooney , J. Bone Miner. Res. 2005, 20, 848. 15824858 10.1359/JBMR.041226

[62] M. M. Martino , F. Tortelli , M. Mochizuki , S. Traub , D. Ben‐David , G. A. Kuhn , R. Müller , E. Livne , S. A. Eming , J. A. Hubbell , Sci. Transl. Med. 2011, 3, 100ra89. 10.1126/scitranslmed.3002614 21918106

[63] M. M. Martino , P. S. Briquez , E. Güç , F. Tortelli , W. W. Kilarski , S. Metzger , J. J. Rice , G. A. Kuhn , R. Müller , M. A. Swartz , J. A. Hubbell , Science 2014, 343, 885. 24558160 10.1126/science.1247663

[64] P. S. Briquez , H.‐M. Tsai , E. A. Watkins , J. A. Hubbell , Sci. Adv. 2021, 7, abh4302. 10.1126/sciadv.abh4302 PMC8195475 34117071

[65] M. Mochizuki , E. Güç , A. J. Park , Z. Julier , P. S. Briquez , G. A. Kuhn , R. Müller , M. A. Swartz , J. A. Hubbell , M. M. Martino , Nat. Biomed. Eng. 2019, 4, 463. 31685999 10.1038/s41551-019-0469-1

[66] A. Grosso , A. Lunger , M. G. Burger , P. S. Briquez , F. Mai , J. A. Hubbell , D. J. Schaefer , A. Banfi , N. D. Maggio , Npj Regen. Med. 2023, 8, 15. 36914692 10.1038/s41536-023-00288-1 PMC10011536

[67] R.‐Z. Lin , R. Moreno‐Luna , R. Muñoz‐Hernandez , D. Li , S.‐C. S. Jaminet , A. K. Greene , J. M. Melero‐Martin , Angiogenesis 2013, 16, 735. 23636611 10.1007/s10456-013-9350-0 PMC3762916

