# Supplementary material for: Human Bone‐Derived Endothelial Cells Mediate Bone Regeneration via Distinct Expression of KIT Ligand
Source: Adv Sci (Weinh). 2025 Jun 23;12(35):e14194. doi: 10.1002/advs.202414194 (PMC12463118; doi:10.1002/advs.202414194)
Supplement: Supplementary file 1 — Supporting Information [file ADVS-12-e14194-s002.pdf]

ADVANCED SCIENCE
Open Access

Supporting Information

for *Adv. Sci.*, DOI 10.1002/advs.202414194

Human Bone-Derived Endothelial Cells Mediate Bone Regeneration via Distinct Expression of KIT Ligand

*Xiang Li, Hwan D. Kim, Allen C. Luo, Liyan Gong, Yonglin Zhu, Chin Nien Lee, Xuechong Hong, Christopher L. Sudduth, Michal Ad, Young-Hyeon An, Mihn Jeong Park, Do-Gyoon Kim, Arin K. Greene, Bonnie L. Padwa, Nathaniel S. Hwang, Ruei-Zeng Lin and Juan M. Melero-Martin**

Supporting Information

Human Bone-Derived Endothelial Cells Mediate Bone Regeneration via Distinct Expression of KIT Ligand

Xiang Li†, Hwan D. Kim†, Allen C. Luo, Liyan Gong, Yonglin Zhu, Chin Nien Lee, Xuechong Hong, Christopher Sudduth, Michal Ad, Young-Hyeon An, Mihn Jeong Park, Do-Gyoon Kim, Arin K. Greene, Bonnie L. Padwa, Nathaniel S. Hwang, Ruei-Zeng Lin, Juan M. Melero-Martin*

Table of Contents:

• **Figure S1.** Characterization of human trabecular bone-derived endothelial cells (b-ECs).

• **Figure S2.** Bone matrix mineralization and mechanical properties of ectopic grafts containing b-ECs.

• **Figure S3.** Histological analysis of bone matrix deposition in ectopic grafts containing b-ECs.

• **Figure S4.** Osteogenic differentiation and bone matrix mineralization of orthotopic grafts in calvarial defects.

• **Figure S5.** Histological evaluation of bone matrix deposition in ectopic grafts with KITLG-modified ECs.

• **Figure S6.** Control experiments evaluating KITLG modulation in b-ECs and ECFCs.

• **Figure S7.** Isolation and characterization of human trabecular bone-derived hematopoietic progenitor cells.

• **Figure S8.** Validation of DAPT-mediated inhibition of Notch signaling in the osteogenic co-culture model.

• **Figure S9.** Notch signaling is required during both HPC activation and subsequent osteogenic induction of bm-MSCs.

• **Table S1.** Sequences of primers used for quantitative RT-PCR.

• **Table S2.** Antibodies used in the study.

• **Dataset S1.** Counts per million (CPM) RNA-seq data for b-ECs, wat-ECS, and ECFCs.

• **Video S1.** μCT evaluation at Week 8 of a representative graft containing b-ECs.

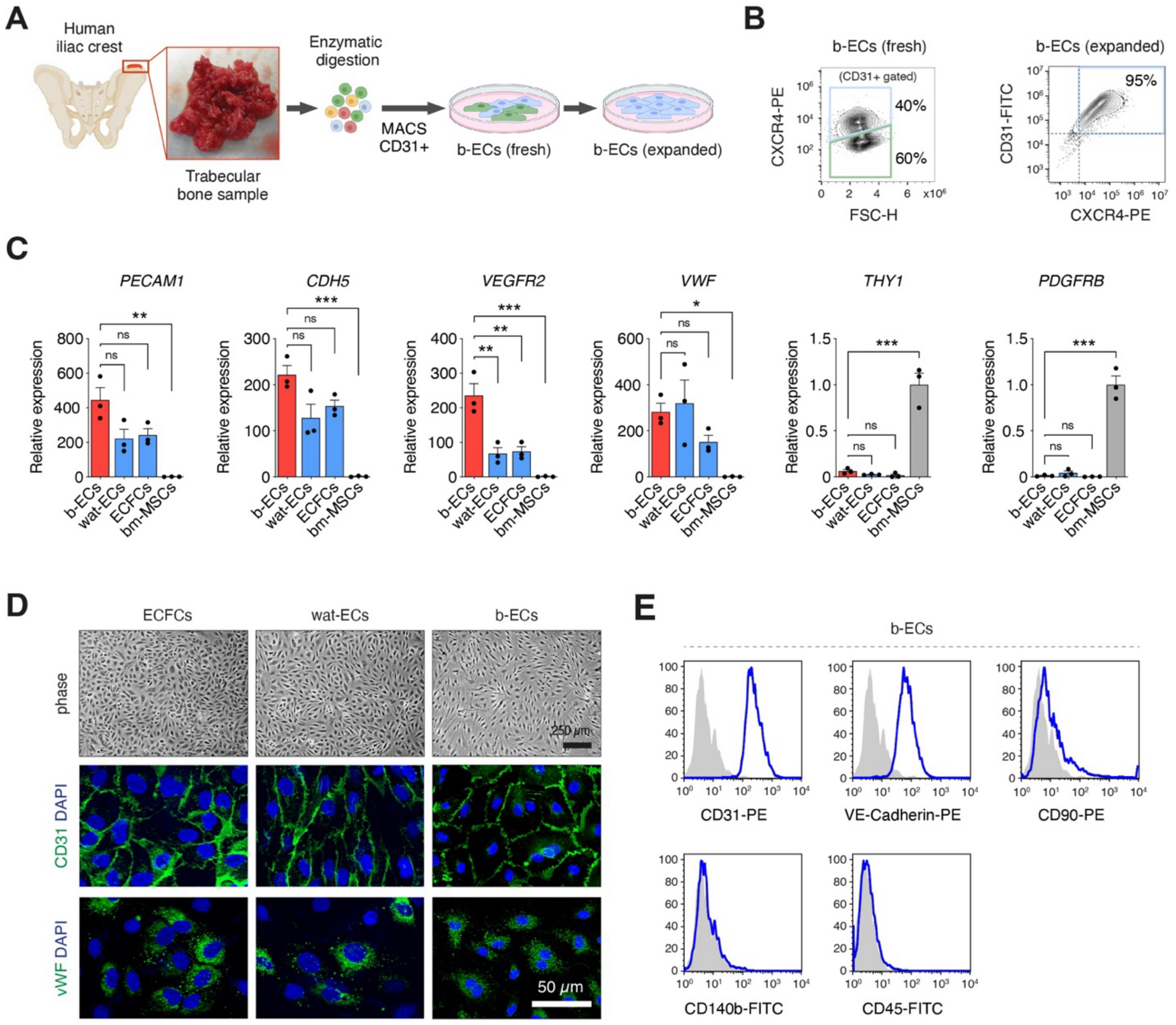

Figure S1. Characterization of human trabecular bone-derived endothelial cells (b-ECs). (**A**) Schematic illustrating the enzymatic digestion of trabecular bone samples obtained from the human iliac crest, leading to the isolation and culture expansion of CD31+ sorted b-ECs. (**B**) Flow cytometry analysis displaying CXCR4 and CD31 expression in freshly isolated versus culture-expanded b-ECs. (**C**) qPCR analysis of endothelial (*PECAM1, CDH5, VEGFR2, VWF*) and mesenchymal (*THY1* and *PDGFRB*) cell markers in b-ECs compared to white adipose tissue ECs (wat-ECs), endothelial colony-forming cells (ECFCs), and bone marrow-derived mesenchymal stem cells (bm-MSCs) (n=3; *P<0.05, **P<0.01, ***P<0.001, ns=not significant). (**D**) Representative phase-contrast and immunofluorescence images of ECFCs, wat-ECs, and b-ECs stained for CD31 and vWF. Scale bar: 250 µm and 50 µm. (**E**) Flow cytometry histograms (blue) of b-ECs show expression of CD31 and VE-Cadherin and lack of expression of CD90, CD140b, and CD45, with grey histograms representing isotype control. Data are mean ± s.e.m. (**C**). *n* are biological replicates (**C**). Statistics are one-way ANOVA with Bonferroni's post-test analysis (**C**). Panel A was partially created with BioRender.com.

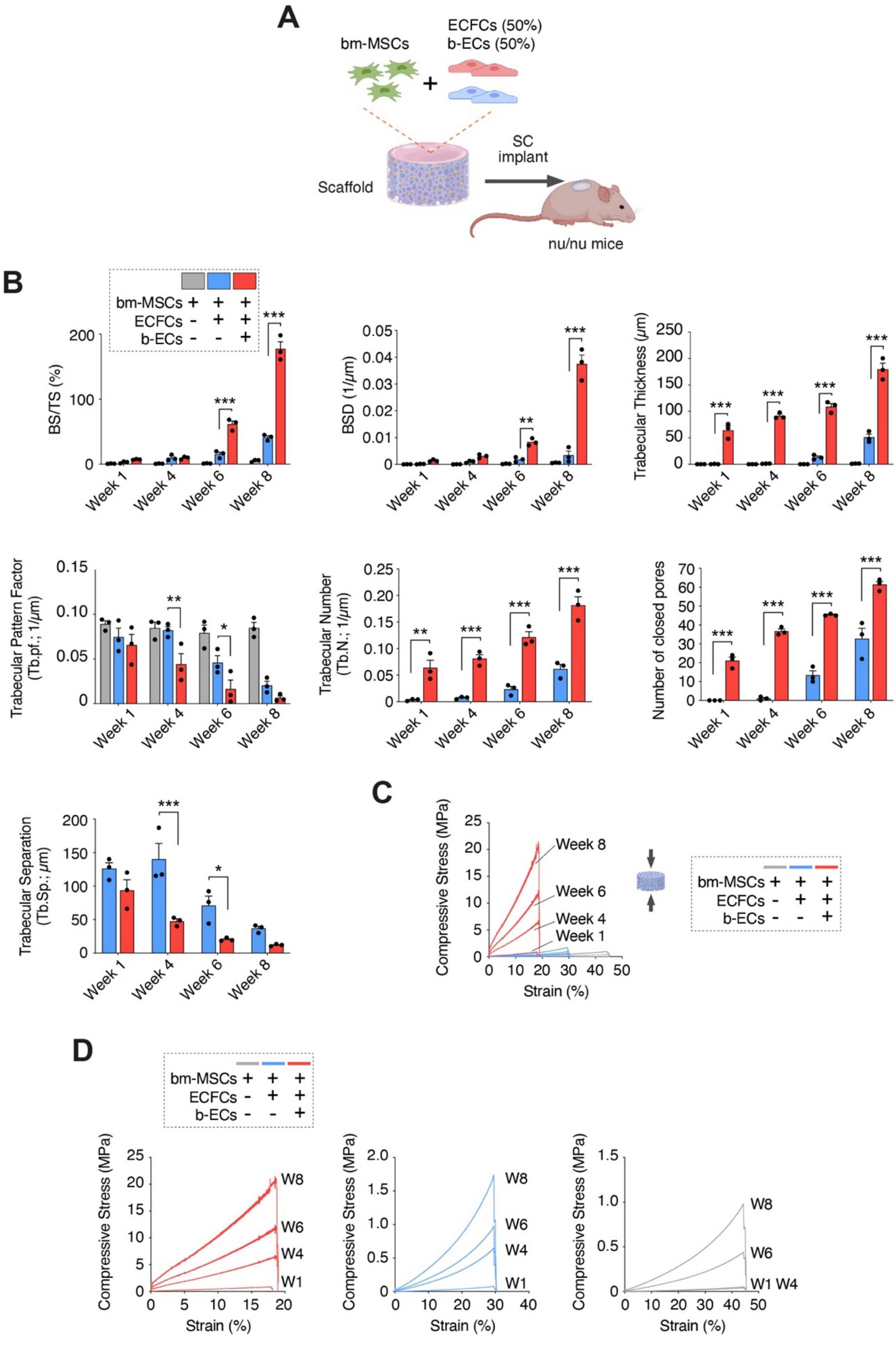

Figure S2. Bone matrix mineralization and mechanical properties of ectopic grafts containing b-ECs. (**A**) Illustration of the subcutaneous implantation of scaffolds laden with bm-

MSCs and either ECFCs alone or in combination with b-ECs into nu/nu mice. (**B**) Graphical representation of μCT analyses of bone surface area to total surface area (BS/TS), bone surface density (BSD), trabecular thickness, trabecular pattern factor, trabecular number, the number of closed pores, and trabecular separation at Weeks 1, 4, 6, and 8 post-implantation (n=3; *P<0.05, **P<0.01, ***P<0.001). (**C**) Combined compressive stress-strain curves for all scaffold types over time. (**D**) Individual compressive stress-strain curves at designated time points (W, Week), indicating the progressive mechanical strength of the grafts. All data are mean ± s.e.m. (**B**). *n* are biological replicates (**B**). Statistics are one-way ANOVA with Bonferroni's post-test analysis (**B**). Panel A was partially created with BioRender.com.

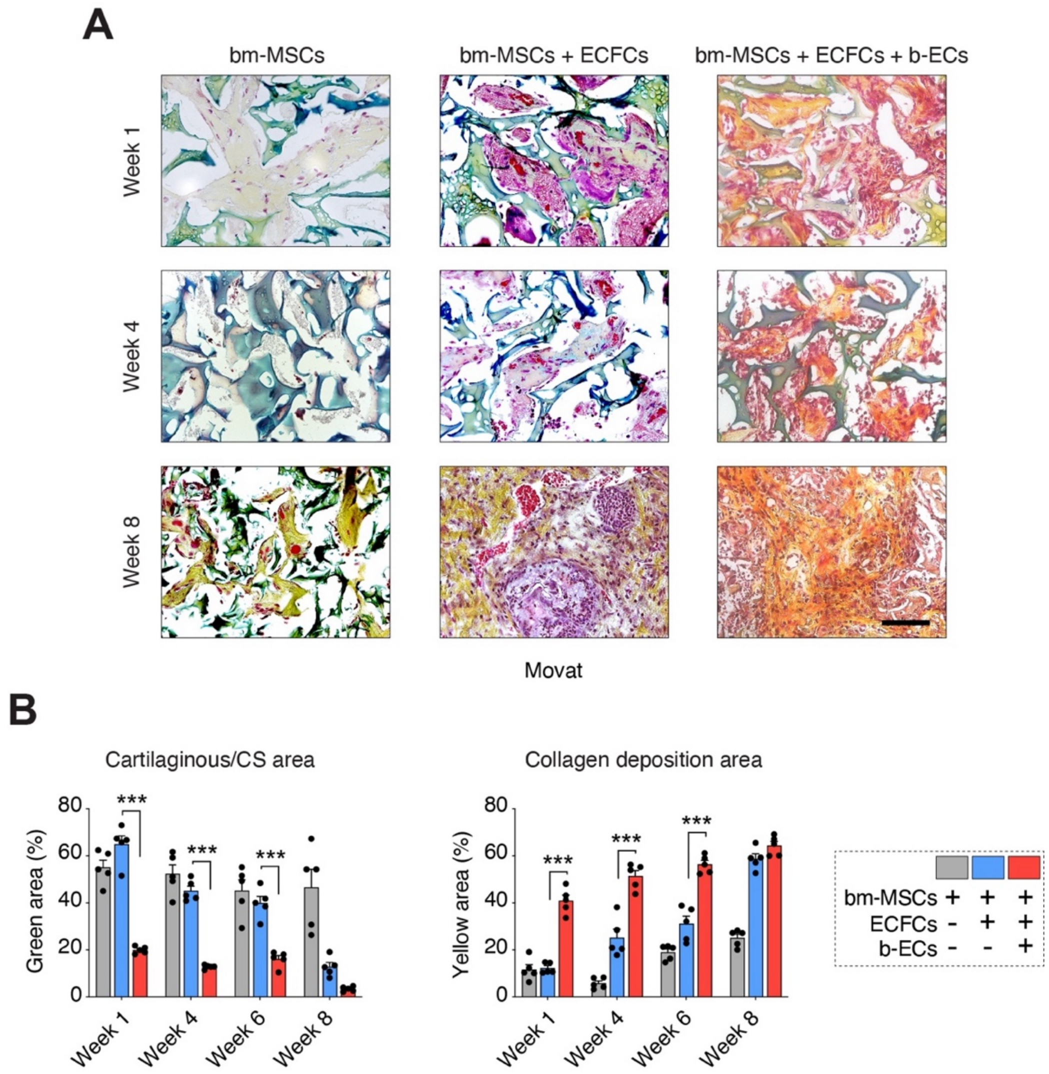

Figure S3. Histological analysis of bone matrix deposition in ectopic grafts containing b-ECs. (**A**) Movat pentachrome staining of grafts containing bm-MSCs and either ECFCs alone or in combination with b-ECs at Weeks 1, 4, and 8 post-implantation. Green indicates cartilaginous/chondroitin sulfate areas, and yellow indicates collagen deposition. Scale bar: 200 µm. (**B**) Quantitative analysis of the green cartilaginous/CS area and the yellow collagen deposition area from the Movat pentachrome staining at each time point, indicating progressive matrix maturation and mineralization in b-EC containing grafts (n=5; ***P<0.001). All data are mean ± s.e.m. (**B**). *n* are biological replicates (**B**). Statistics are one-way ANOVA with Bonferroni's post-test analysis (**B**).

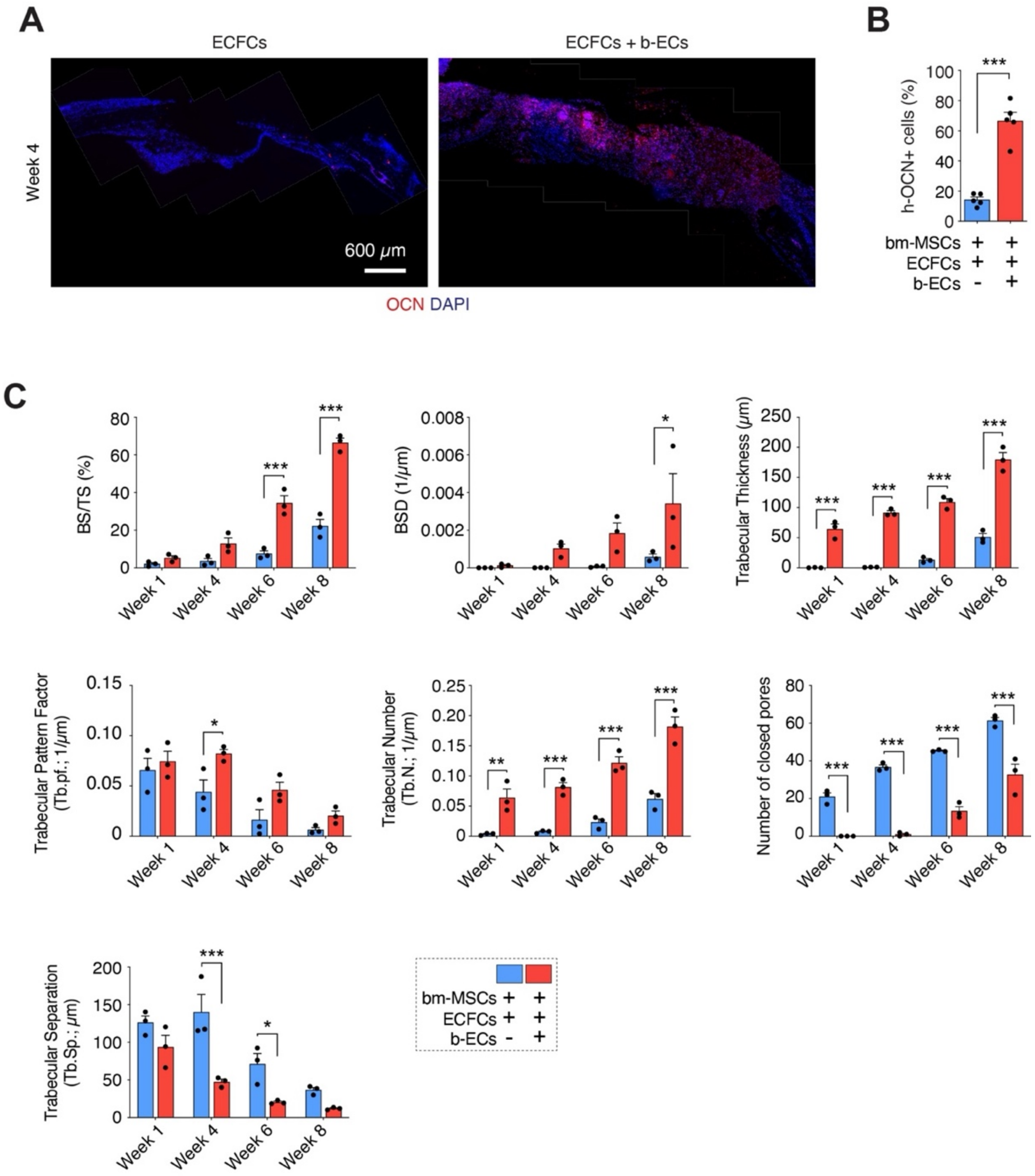

Figure S4. Osteogenic differentiation and bone matrix mineralization of orthotopic grafts in calvarial defects. (**A**) Implantation of scaffolds laden with bm-MSCs and either ECFCs alone or in combination with b-ECs into critical-size calvarial defects in nu/nu mice. Immunofluorescent staining for osteocalcin (OCN) at Week 4. Scale bar: 600 µm. (**B**) Quantification of human OCN+ cells within the grafts indicates enhanced osteogenic activity in b-EC-containing grafts (n=5; ***P<0.001). (**C**) Detailed quantitative µCT analysis of the grafts, including bone surface area per total surface area (BS/TS), bone surface density (BSD), trabecular thickness, trabecular pattern factor, trabecular number, number of closed pores, and trabecular separation, showing significant improvement in structural parameters in b-EC-containing grafts over time (n=3; *P<0.05, **P<0.01, ***P<0.001). All data are mean ± s.e.m. (**B**, **C**). *n* are biological replicates (**B**, **C**). Statistics are unpaired two-tailed Student's t-tests (**B**) and one-way ANOVA with Bonferroni's post-test analysis (**C**).

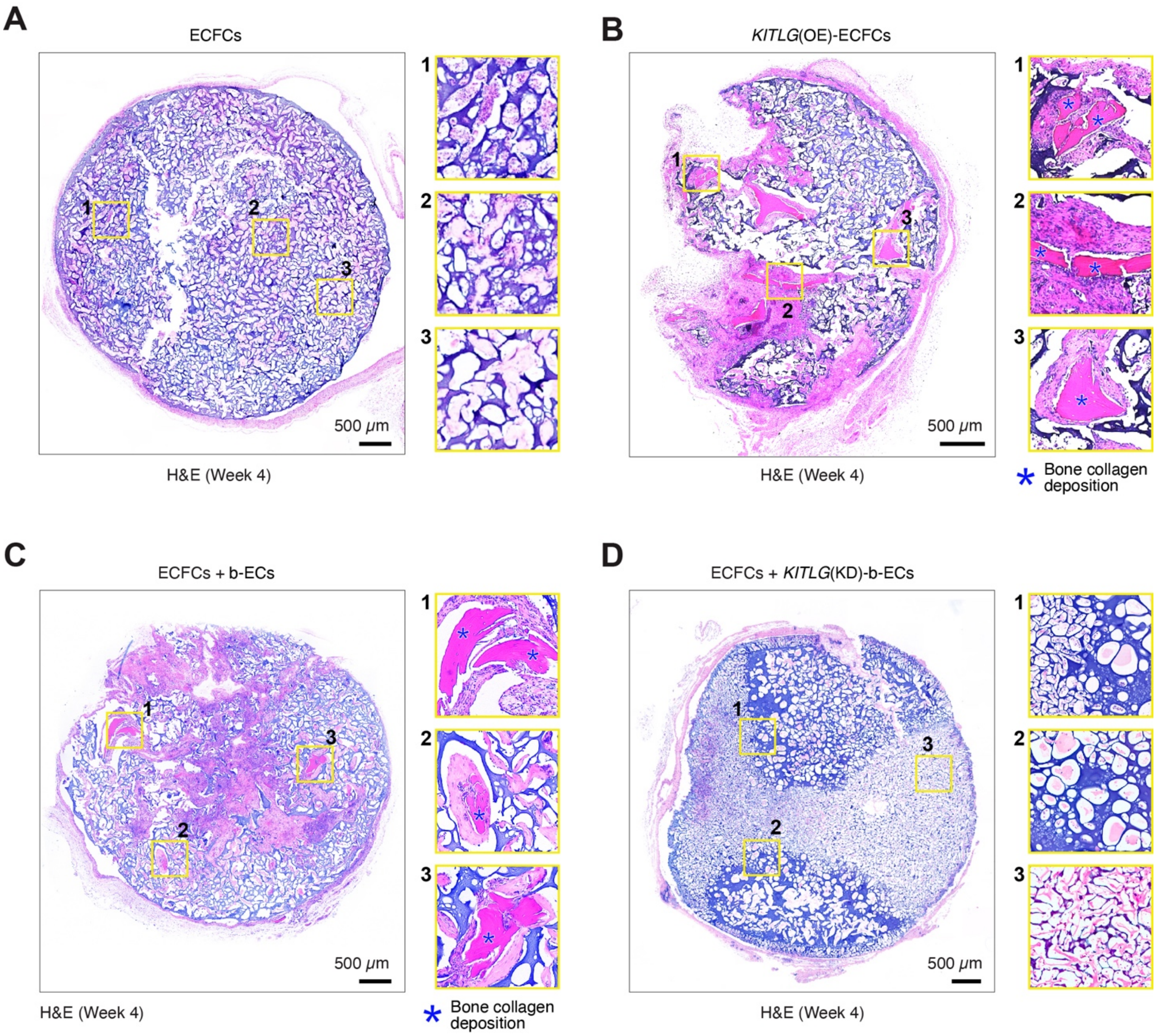

Figure S5. Histological evaluation of bone matrix deposition in ectopic grafts with KITLG-modified ECs. (**A**) (A–D) Representative low-magnification H&E staining of explanted grafts at 4 weeks post-implantation from the indicated groups: (**A**) ECFCs, (**B**) KITLG(OE)-ECFCs, (**C**) ECFCs + b-ECs, and (**D**) ECFCs + KITLG(KD)-b-ECs. Yellow boxes indicate the locations of three randomly selected high-magnification regions, which are displayed adjacent to each corresponding full-graft image. Blue asterisks highlight areas of bone collagen deposition where present. Scale bar: 500 μm.

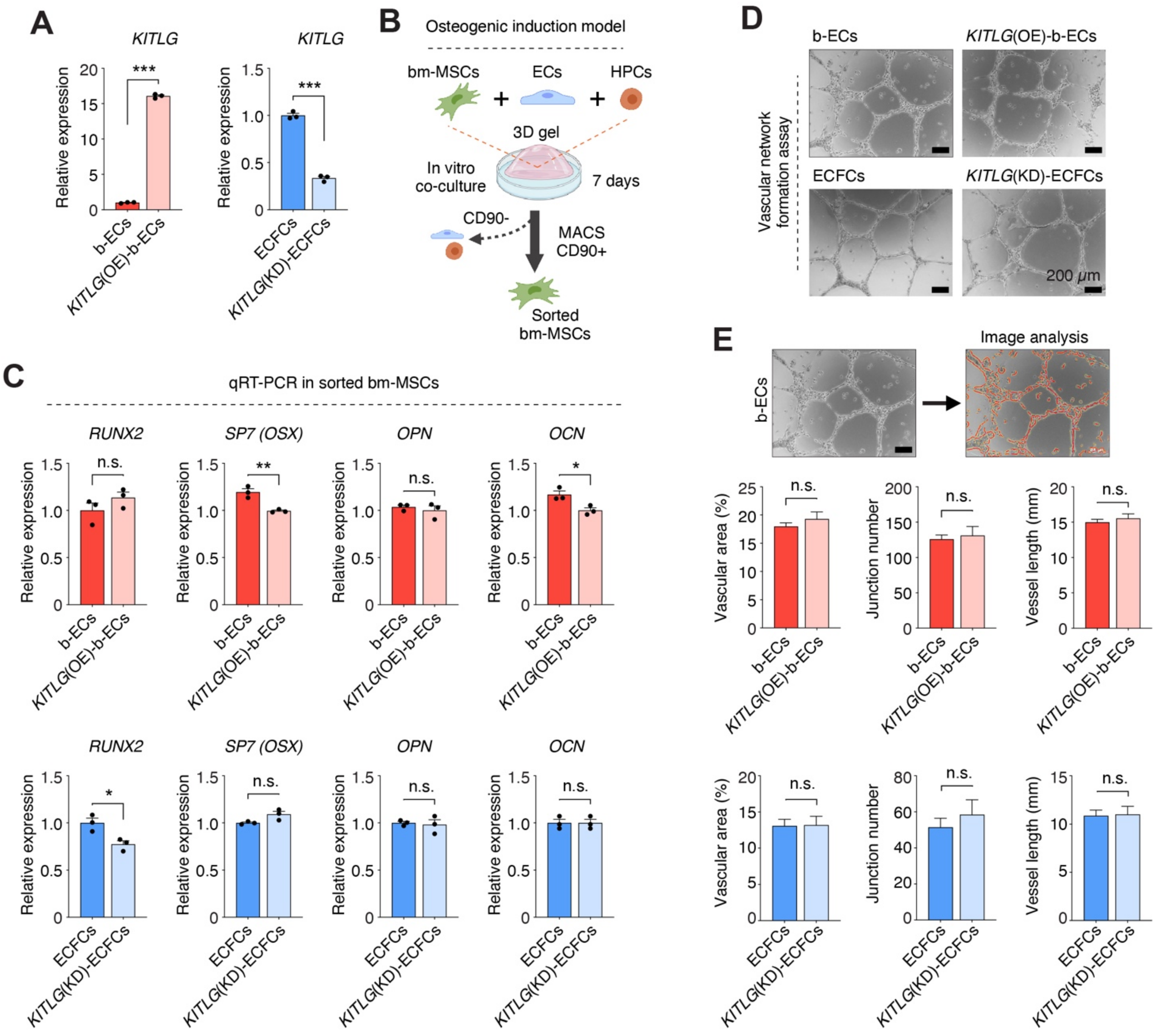

Figure S6. Control experiments evaluating KITLG modulation in b-ECs and ECFCs. (**A**) qRT-PCR validation of KITLG overexpression in b-ECs [KITLG(OE)-b-ECs] and knockdown in ECFCs [KITLG(KD)-ECFCs] using lentivirus. (**B**) Schematic of the 3D in vitro osteogenic co-culture model: bm-MSCs were co-cultured with ECs and c-Kit^{+} HPCs in a collagen/fibrin hydrogel for 7 days, followed by magnetic sorting of CD90^{+} bm-MSCs. (**C**) qRT-PCR analysis of osteogenic marker expression (*RUNX2*, *SP7/OSX*, *OPN*, and *OCN*) in sorted bm-MSCs comparing b-ECs vs. KITLG(OE)-b-ECs (top) and ECFCs vs. KITLG(KD)-ECFCs (bottom) (n=3). (**D**) Representative phase-contrast images from Matrigel-based tube formation assays showing vascular network formation by b-ECs, KITLG(OE)-b-ECs, ECFCs, and KITLG(KD)-ECFCs. Scale bar: 200 μm. (**E**) Quantification of vascular area, junction number, and vessel length between each respective control and KITLG-modified EC group, analyzed using the AngioTool 2.0 plugin in ImageJ. Data are mean ± s.e.m. (n=3); *P<0.05, **P<0.01, ***P<0.001; n.s., not significant. *n* are biological replicates. Statistics are unpaired two-tailed Student's t-tests (A, C, E). Panel B was partially created with BioRender.com.

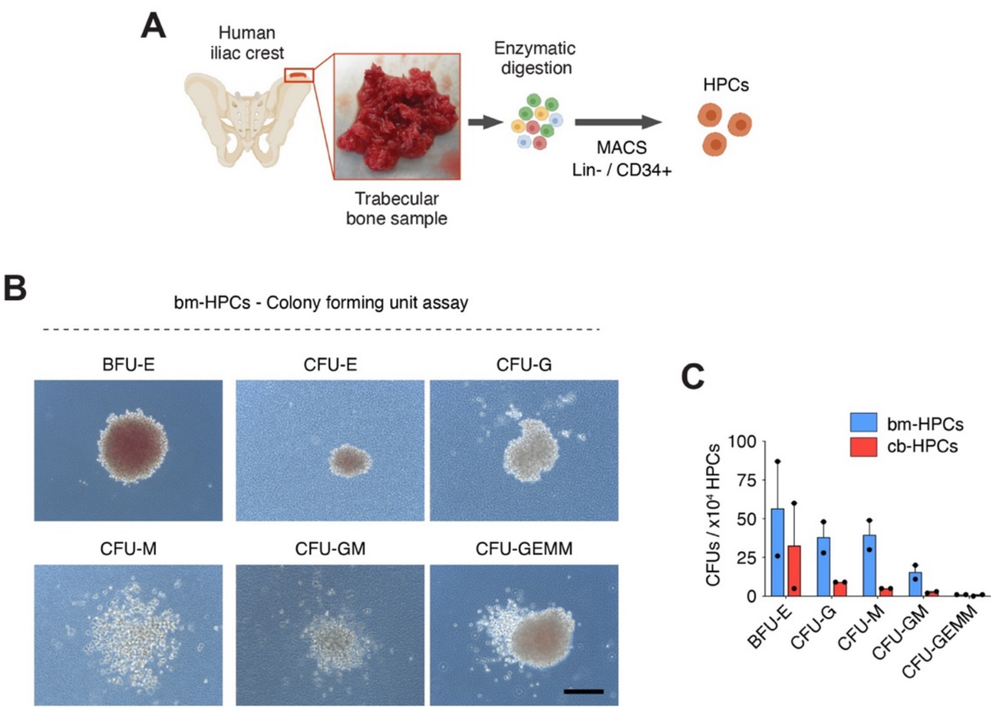

Figure S7. Isolation and characterization of human trabecular bone-derived hematopoietic progenitor cells. (**A**) Schematic representing the extraction and isolation process for Lin-/CD34+ hematopoietic progenitor cells (HPCs) from human iliac crest trabecular bone samples. (**B**) Colony-forming unit assay images depicting various colony types derived from bone marrow HPCs (bm-HPCs). Colonies were categorized as erythroid (BFU-E, CFU-E), myeloid (CFU-G/M/GM), and mixed (CFU-GEMM). Scale bar: 500 µm. (**C**) Quantitative analysis of colony-forming units per 10^4 HPCs from bone marrow (bm-HPCs) and cord blood (cb-HPCs), including burst-forming unit-erythroid (BFU-E), granulocyte (CFU-G), macrophage (CFU-M), granulocyte-macrophage (CFU-GM), and granulocyte, erythrocyte, monocyte, megakaryocyte (CFU-GEMM). Data are mean ± s.e.m. (n=2). *n* are biological replicates. Panel A was partially created with BioRender.com.

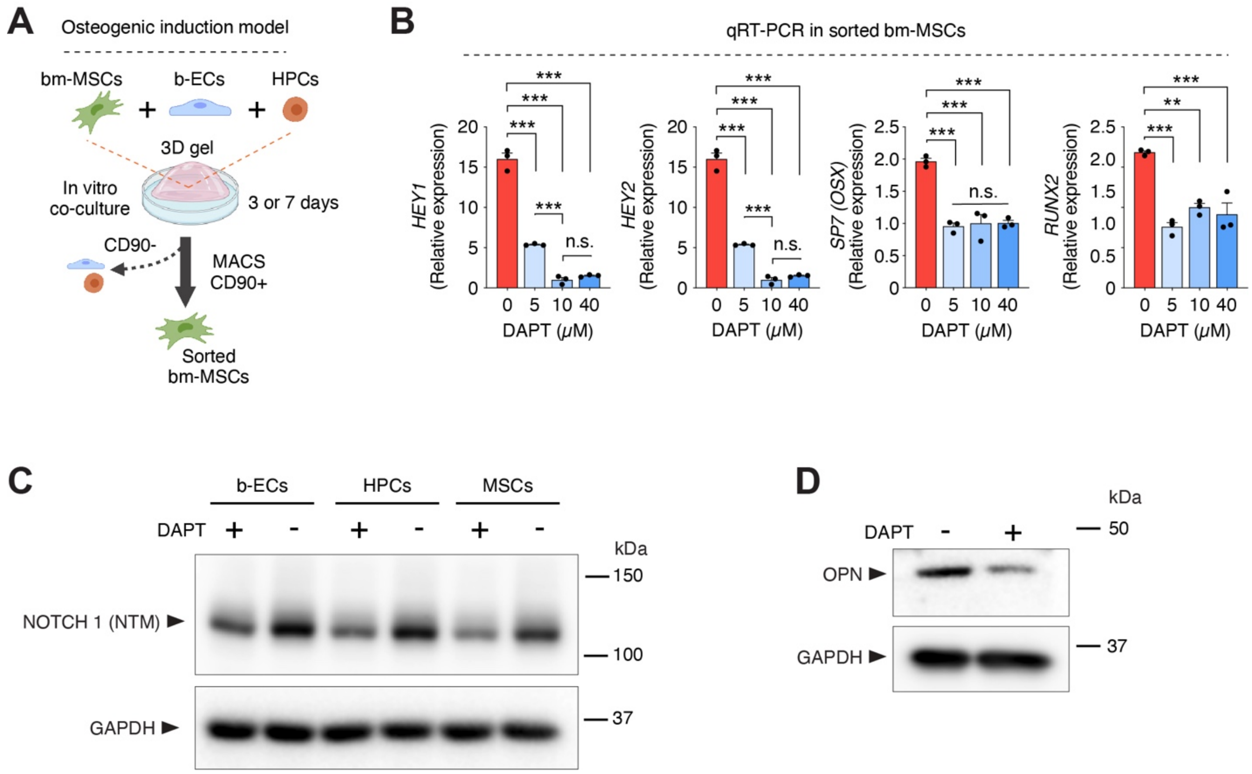

Figure S8. Validation of DAPT-mediated inhibition of Notch signaling in the osteogenic co-culture model. (**A**) Schematic of the 3D in vitro osteogenic co-culture model used for DAPT titration: bm-MSCs were co-cultured with b-ECs and c-Kit^{+} HPCs in a collagen/fibrin hydrogel for either 3 or 7 days, depending on the experiment, followed by magnetic sorting of CD90^{+} bm-MSCs. (**B**) qRT-PCR analysis of canonical Notch target genes (*HEY1*, *HEY2*) and osteogenic markers (*SP7/OSX*, *RUNX2*) in sorted bm-MSCs following treatment with increasing doses of DAPT (5, 10, 40 μM) for 3 days (n=3). (**C**) Western blot analysis of NOTCH1 (NTM) protein level in b-ECs, HPCs, and bm-MSCs treated with (+) or without (–) DAPT (10 μM), confirming effective inhibition of Notch signaling across all three cell types. GAPDH was used as a loading control. (**D**) Western blot analysis of osteopontin (OPN) expression in CD90^{+} bm-MSCs isolated from 3D co-cultures treated with or without DAPT (10 μM) for 7 days, showing reduced osteogenic marker expression upon Notch inhibition. GAPDH was used as a loading control. Data are mean ± s.e.m. (B). *n* are biological replicates (B). Statistics are one-way ANOVA with Bonferroni's post-test analysis (B). Panel A was partially created with BioRender.com.

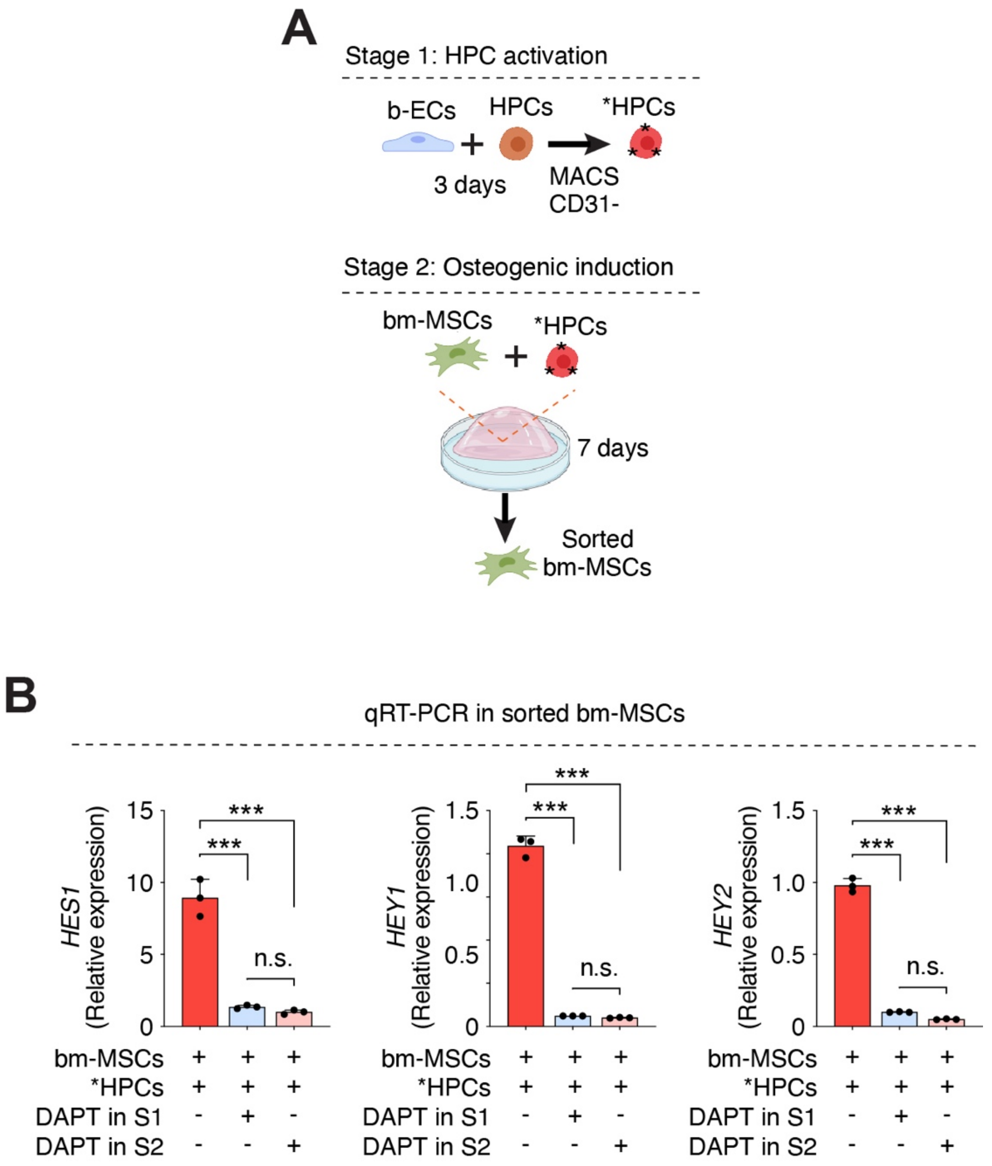

Figure S9. Notch signaling is required during both HPC activation and subsequent osteogenic induction of bm-MSCs. (**A**) Schematic of the two-stage 3D co-culture model used to dissect the role of Notch signaling: Stage 1 involved b-EC-mediated activation of HPCs for 3 days, and Stage 2 involved co-culture of activated *HPCs with bm-MSCs for 7 days. DAPT was selectively applied during either Stage 1 or Stage 2 to inhibit Notch signaling at specific points. (**B**) qRT-PCR analysis of canonical Notch target gene expression (*HES1*, *HEY1*, *HEY2*) in sorted bm-MSCs after co-culture. Inhibition of Notch signaling during Stage 1 impaired the ability of HPCs to subsequently activate Notch targets in bm-MSCs, while inhibition during Stage 2 suppressed Notch activation directly in bm-MSCs. Data are mean ± s.e.m. (n=3); ***P<0.001; n.s., not significant. *n* are biological replicates. Statistics are one-way ANOVA with Bonferroni's post-test analysis (B). Panel A was partially created with BioRender.com.

Table S1. Sequences of primers used for quantitative RT-PCR.

Gene	Forward (5’ to 3’)	Reverse (3’ to 5’)
ALPL	GTTGCCAAGCTGGGAAGAACAC	CCCACCCCGCTATTCCAAAC
RUNX2	CGGTCTCCTTCCAGGATGGT	GCTTCCGGTCAGCGTCAACA
Osteocalcin (OCN)	GACAAAGCCTTCATGTCCAAG	AAAGCCGAGCTGCCAGAGTTT
Osteopontin (OPN)	CGAGGAGTTGAATGGTGCATAC	CATCCAGCTGACTCGTTTCATAA
Osterix (OSX, SP7)	TGAGCTGGAGCGTCATGTG	GGTGGTCGCTTCGGGTAAA
Collagen I	AACCCGAGGTATGCTTGATCT	CCAGTTCTTCATTGCATTGC
GAPDH	AGGAGTATATGCCCGACGTG	TCGTCCACATCCACACTGTT
KITLG	GCCAGCTCCCTTAGGAATGAC	TAAGGCTCCAAAAGCAAAGCC
PECAM1/CD31	CACCTGGCCCAGGAGTTTC	AGTACACAGCCTTGTTGCCATG
CDH5/VE-Cadherin	GAACCCAAGATGTGGCCTTTAG	GATGTGACAACAGCGAGGTGTAA
VEGFR2	ATCCAGTGGGCTGATGACCAAGAA	ACCAGAGATTCCATGCCACTTCCA
VWF	GTCGAGCTGCACAGTGACATG	GCACCATAAACGTTGACTTCCA
THY1/CD90	GCCTAACGGCCTGCCTAGT	GGGTGAACTGCTGGTATTCTCAT
PDGFRB	TCTTTGTGCCAGATCCCACC	AGTGCAACGTCCCCTTTCTT
eNOS	AGATCTCCGCCTCGCTCAT	GTCTCGGAGCCATACAGGATTG
Sox9	CCCCAACAGATCGCCTACAG	TCTGGTGGTCGGTGTAGTCGTA
HES1	TCAGCGAGTGCATGAACGA	TTGATCTGGGTCATGCAGTTG
HEY1	TTTCAAGAAACACTTCTTGTTCCA	TGTCTAAATTGATTTGATTTTGCCG
HEY2	AGCCCTGTTGAGGAGAACCATACTA	ACCACTGAGATTGTGTCTTGTGA

All primers were designed to specifically recognize human, but not murine, genes.

Table S2. Antibodies used in the study.

Antibody	Vendor	Cat number	Dilution*
Mouse anti-human Vimentin (V9)	Abcam	Ab8069	1:200 (IF)
Rabbit anti-Osterix (OSX)	Abcam	ab22552	1:200 (IF)
Goat anti-human/mouse CD117/c-Kit	Novus Biologicals	AF1356	1:50 (IF, IHC)
Mouse anti-human CD31 (JC70A, human specific)	Agilent	M082329-2	1:200 (IF)
Mouse anti-human CD34 Antibody	BioLegend	343608	1:200 (IF)
Rabbit anti-human Osteocalcin (OCN)	Proteintech	23418-1-AP	1:200 (IF)
Rat anti-human RUNX2/CBFA1 (232902)	R&D Systems	MAB2006	1:200 (IF)
Rabbit anti-human Von Willebrand Factor (vWF)	DAKO	A0082	1:200 (IF)
Mouse anti-human Osteocalcin (OCN)	R&D Systems	MAB1419-SP	1:200 (IF)
Rabbit anti-α smooth muscle actin (1A4)	Sigma-Aldrich	A2547	1:300 (IF)
Rhodamine labeled Ulex Europaeus Agglutinin I (UEA I, human specific)	Vector Laboratories	RL-1062	1:100 (IF)
PE anti-human CD90 (Thy1)	BioLegend	328109	1:100 (MACS)
Texas Red -conjugated horse anti-mouse IgG	Vector Laboratories	TI-2000	1:200 (IF)
FITC-conjugated horse anti-mouse IgG	Vector Laboratories	FI-2000	1:200 (IF)
Peroxidase-conjugated horse anti-mouse IgG	Vector Laboratories	PI-2000	1:200 (IHC)
Texas Red-conjugated goat anti-rabbit IgG	Vector Laboratories	TI-1000	1:200 (IF)
FITC-conjugated goat anti-rabbit IgG	Vector Laboratories	FI-5000	1:200 (IF)
Donkey anti-Rat IgG	ThermoFisher	A78946	1:200 (IF)
Donkey anti-Goat IgG	ThermoFisher	A32814	1:200 (IF)
Notch1 (D1E11) XP® Rabbit mAb	Cell Signaling Technology	3608S	1:1000 (WB)

Human Osteopontin/OPN Antibody	R&D Systems	MAB14331-100	1:1000 (WB)
Anti-GAPDH antibody	Millipore Sigma	G9545-100UL	1:1000 (WB)
Anti-Rabbit-HRP	Kindle Biosciences	R1004	1:1000 (WB)
Anti-Mouse-HRP	Kindle Biosciences	R1004	1:1000 (WB)

* IHC: immunohistochemistry staining; IF: immunofluorescence staining; MACS: magnetic-activated cell sorting; WB: western blot.

Dataset S1. Counts per million (CPM) RNA-seq data for b-ECs, wat-ECS, and ECFCs.

Video S1. μCT evaluation at Week 8 of a representative graft containing b-ECs.
